# Computational Approaches to Neurological Disorder Diagnosis: An In-Depth Review of Current Methods and Future Prospects

**DOI:** 10.2174/0115734056404224251026110800

**Published:** 2025-11-21

**Authors:** Kabita Patel, T. Sarathamani, Kavitha Kothandasamy, Prabira Kumar Sethy, Santi Kumari Behera, Aziz Nanthaamornphong

**Affiliations:** 1 Department of CSE, SUIIT, Sambalpur University, Jyoti Vihar, Burla, Sambalpur, India; 2 Department of Computer Science and Engineering-AI, Brainware University, Kolkata, India; 3 School of Computing, Department of AI and ML, Mohan Babu University, Tirupati, AP, India; 4 Department of Electronics, Sambalpur University, Burla, Odisha, Sambalpur, India; 5 Department of CSE, VSSUT Burla, Odisha, Sambalpur, India; 6 College of Computing, Prince of Songkla University, Phuket, Thailand

**Keywords:** Brain diseases, Machine learning, Deep learning, Alzheimer’s disease, Parkinson’s disease, Epilepsy, Huntington’s disease, Amyotrophic lateral sclerosis

## Abstract

The rapid advancement of computational technologies has significantly transformed medical diagnostics, particularly in the realm of neurological disorders. This review provides a comprehensive analysis of the current computational approaches employed for the diagnosis of five major neurological disorders: Alzheimer’s disease, Parkinson’s disease, Epilepsy, Huntington’s disease, and Amyotrophic Lateral Sclerosis. By evaluating 140 peer-reviewed studies, we explored a diverse array of diagnostic methods, including machine learning algorithms, neuroimaging techniques, and electrophysiological signal analysis. Our review highlights the efficacy, accuracy, and limitations of these diagnostic methods, emphasizing their role in early detection and differential diagnosis. Furthermore, we discuss the integration of multimodal data and the potential of emerging technologies such as deep learning and artificial intelligence to enhance diagnostic practices. We also address the current challenges in clinical implementation and propose future research directions to improve diagnostic precision and patient outcomes. This review aims to serve as a valuable resource for researchers, clinicians, and stakeholders in the field of neurodiagnostics, fostering a deeper understanding of computational methodologies that shape the future of neurological disorder diagnosis.

## 1 INTRODUCTION

Brain diseases such as Alzheimer's Disease (AD), Parkinson's Disease (PD), Epilepsy, Huntington's Disease (HD), and Amyotrophic Lateral Sclerosis (ALS) present significant challenges to healthcare systems worldwide [1]. These disorders not only impact the quality of life of millions of individuals but also impose substantial economic burdens on society. Traditionally, the diagnosis of these diseases has relied heavily on clinical evaluations, imaging studies, and genetic testing. However, the complexity and heterogeneity of brain diseases necessitate more sophisticated and precise diagnostic tools [2].

Recent advances in computational technologies have ushered in a new era of medical diagnostics, providing powerful tools to aid in the detection, classification, and monitoring of brain diseases [3]. The application of computational methods, ranging from Machine Learning (ML) algorithms to neuroimaging analysis, has shown promise in improving the diagnostic accuracy and enabling early detection, which are crucial for effective treatment and management [4].

The primary purpose of this review was to provide an in-depth analysis of the current state of computational approaches for the diagnosis of five major brain diseases: AD, PD, Epilepsy, HD, and ALS. By synthesizing the findings from 140 peer-reviewed studies, we aimed to:

- Evaluate Efficacy: Assess the effectiveness of various computational methods in diagnosing brain diseases, highlighting their strengths and limitations.
- Explore multimodal integration: Examine the integration of multimodal data sources, such as neuroimaging and electrophysiological signals, to enhance diagnostic precision.
- Identify Emerging Technologies: Discuss the potential of emerging computational technologies, including DL and artificial intelligence, to revolutionize the field of neurodiagnostics.
- Highlight Challenges and Future Directions: Address the current challenges in clinical implementation and propose future research directions to overcome these barriers and improve patient outcomes.

Computational approaches in brain disease diagnosis encompass a broad spectrum of methods that leverage computational power and advanced algorithms to analyze complex biological data [5]. These methods include, but are not limited to, ML, DL, neuroimaging, and signal processing. The importance of these approaches lies in their ability to handle large datasets, uncover hidden patterns, and provide quantitative insights that are often beyond the scope of the traditional diagnostic methods [6].

For instance, ML algorithms can be trained to recognize subtle patterns in brain imaging data that are indicative of early-stage disease, whereas DL models can analyze vast amounts of genetic and proteomic data to identify biomarkers associated with brain disorders [7]. Neuroimaging techniques, enhanced by computational analysis, enable detailed visualization of brain structure and function and facilitate the early detection of pathological changes.

In summary, computational approaches hold significant promise for advancing the diagnosis of brain diseases by providing accurate, early, and personalized diagnostic capabilities. This review sought to elucidate the current landscape of these approaches and their potential to transform neurodiagnostics, ultimately aiming to improve the quality of lifeofindividuals affected bythesedebilitating conditions. Figure (**1**) illustrates the overview Structural Diagram of the Review Framework.

## 2 METHODOLOGY

To ensure a comprehensive review of current computational approaches for brain disease diagnosis, a systematic literature search was conducted. The inclusion criteria were studies relevant to the use of computational methods for diagnosing AD, PD, Epilepsy, HD, and ALS. Only peer-reviewed research articles, review papers, and conference proceedings published within the last ten years were considered, and the search was restricted to English-language publications. Studies that were not directly related to computational approaches in brain disease diagnosis, those from non-peer-reviewed sources, or duplicate studies were excluded.

The literature search spanned several major academic databases, including PubMed, IEEE Xplore, ScienceDirect, Web of Science, and Google Scholar. A combination of keywords was employed to ensure comprehensive coverage of the relevant literature. Primary keywords included “Computational approaches,” “Brain disease diagnosis,” “Alzheimer's disease,” “Parkinson's disease,” “Epilepsy,” “Huntington's disease,” “Amyotrophic Lateral Sclerosis,” “Machine learning,” “Deep learning,” “Neuroimaging,” “Electrophysiological signals,” and “Biomarkers.” Boolean operators were used to refine the search results by combining disease-specific terms with computational method terms (*e.g*., “Alzheimer’s disease AND machine learning”). Filters were applied to limit the search to the last 10 years and English-language publications. The reference lists of the relevant articles were also reviewed to identify additional studies that met the inclusion criteria.

Each identified study was initially screened according to its title and abstract to assess its relevance. The full-text articles of potentially relevant studies were reviewed in detail to determine their inclusion in the final analysis. Discrepancies in the selection process were resolved through discussions among the reviewers to ensure consensus. By adhering to this systematic and rigorous methodology, this review aims to provide a thorough and accurate synthesis of the current state of computational approaches for the diagnosis of major brain diseases, offering valuable insights into their efficacy, challenges, and future prospects.

## 3 CURRENT COMPUTATIONAL APPROACHES IN BRAIN DISEASE DIAGNOSIS

Current computational approaches to brain disease diagnosis encompass a range of methodologies, including Machine Learning (ML), neural networks, and data mining. These methods leverage the power of algorithms and computational models to analyze complex datasets, such as neuroimaging, electrophysiological signals, and genetic data. ML techniques, such as supervised and unsupervised learning, excel in pattern recognition and classification tasks and provide high diagnostic accuracy and early disease detection. Neural networks, particularly DL models such as CNNs and RNNs, are adept at handling high-dimensional data and extracting meaningful features from raw data. Data mining techniques, including clustering and anomaly detection, are valuable for identifying hidden patterns and relationships within large datasets. However, these methods have limitations. ML models often require extensive labeled data for training and can suffer from issues related to interpretability. Although powerful, neural networks are computationally intensive and can act as “black boxes,” making their decision-making processes difficult to understand. Data mining techniques require significant data preprocessing and careful interpretation to avoid false associations. Despite these challenges, the integration of these computational approaches continues to enhance their accuracy, efficiency, and early diagnostic capabilities in the field of brain disease diagnosis, offering promising directions for future research and clinical applications. The next section details five types of brain diseases.

## 4 ALZHEIMER’S DISEASE

AD is a degenerative neurological condition that results in a gradual decline in cognitive abilities, such as memory loss, as well as changes in personality and conduct. This condition is the primary cause of dementia and affects millions of elderly individuals worldwide. This disease is characterized by the accumulation of neurofibrillary tangles and amyloid plaques in the brain, leading to neuronal damage and brain atrophy. The necessity of sophisticated diagnostic instruments and research in this field is underscored by the need for prompt identification to effectively manage symptoms and impede progress. Figure (**2**) illustrates healthy brain tissue and the tissue affected by Alzheimer's.

A team of researchers led by Marwa *et al.* is currently developing a state-of-the-art DL-based system specifically designed for the precise diagnosis and classification of various stages of AD [8]. This pipeline employs a rudimentary CNN architecture in conjunction with 2D T1-weighted magnetic resonance brain images. It guarantees a rapid and precise diagnosis by providing a comprehensive categorization of both local and global dimensions. In comparative evaluations, the proposed strategy surpassed the current DL methodologies, obtaining an extraordinary testing accuracy of 99.68% throughout all phases.

This review investigates non-invasive biomarkers that can be used to detect AD at an early stage. This underscores the robust correlation between the brain pathology and these biomarkers. These indicators facilitate prompt treatment, which is essential given the progressive nature of AD and its effects on cognitive function [9].

Therriault *et al.* [10] evaluated the efficacy of immunoassay and mass spectrometry methodologies in the diagnosis of AD by detecting phosphorylated tau (ptau) in Cerebrospinal Fluid (CSF). The results of both methods for ptau217 were comparable; however, the immunoassays demonstrated marginally superior performances for p-tau181 and p-tau231. Immunoassay-based p-tau biomarkers have a modest advantage over MS-based biomarkers. Additional investigations are necessary to ascertain whether simultaneous analysis of numerous biomarkers can compensate for the reduced precision of mass spectrometry in measuring p-tau levels.

The aim of this study, which was conducted by Prasath *et al.* [11], was to detect AD in brain images using a DL algorithm that integrates the Pipelined LeNet (PLN) architecture with fusion techniques. Preprocessing is a procedure to reduce the size and improve the quality of the internal regions of brain MRI images. The PLN algorithm accurately detects AD by analyzing and categorizing the ternary features extracted from the merged image. The efficiency and speed of the proposed system are attributed to its novel PLN architecture, which enables it to achieve high classification accuracy even when dealing with low-resolution images. The Kaggle Alzheimer's Classification Dataset (KACD) exhibits exceptional precision, accuracy, specificity, and sensitivity, as evidenced by its evaluation. Compared to alternative alternatives, the system exhibits superior efficacy by requiring less execution time. The efficacy of the proposed approach was demonstrated by comparing the experimental results with those in the existing literature.

Illakiya *et al.* [12] conducted a review of 103 germane literature to investigate the influence of DL on the identification of AD. A variety of advanced ML techniques, including CNNs, RNNs, and TL, use neuroimaging data, such as PET and MRI. This study emphasizes the importance of the proper diagnosis, segmentation, and classification of AD using radiological criteria. Despite the primary emphasis on DL technologies, there is a lack of research on the impact of various biomarkers on Alzheimer's disease. Articles published in English were also assessed. This study underscores the significance of conducting additional research on the progression from Mild Cognitive Impairment (MCI) to AD using DL models. In addition, it endeavors to identify significant research deficits and constraints.

Vrahatis *et al.* [13] explored the potential of noninvasive biomarkers to identify early-stage AD. This study emphasizes the critical importance of DL techniques and Artificial Intelligence (AI) in this process. This statement underscores the importance of employing AI and DL techniques to analyze large datasets generated by noninvasive procedures, such as imaging, blood monitoring, and wearable sensors. This study underscores the significance of obtaining precise results using these methods, as they have the potential to revolutionize the early diagnosis of AD and resolve the current diagnostic challenges.

In a study conducted by Aberathne *et al.* [14], longitudinal data analysis and ML techniques were employed to investigate early detection of AD using MRI and PET neuroimaging. The advantage lies in the ability to detect minute changes in brain structure and function over time through the application of these advanced methodologies, which allows the prediction of AD progression before clinical symptoms appear. To produce precise and dependable results, the investigation implemented a rigorous research methodology that entailed the acquisition and examination of longitudinal data. These findings underscore the potential of combining ML with MRI and PET neuroimaging to identify the early phases of Alzheimer's disease, thereby opening up new opportunities for the development of more effective diagnostic and therapeutic methods.

Marwa, EL-Geneedy, *et al.* [8] proposed a unique DL method based on MRI that is capable of accurately identifying AD. This study is noteworthy for its innovative approach that integrates DL techniques with MRI data processing to improve the precision of AD diagnosis. The research procedure employed in this study was rigorous and involved the construction and training of deep neural network models using MRI data. The objective was to achieve superior performance in the identification of AD in comparison with our current methods. The findings of this study illustrate the potential of MRI-based DL algorithms to provide physicians with a reliable instrument for the early and accurate diagnosis of AD, thereby facilitating earlier intervention and treatment.

Petti *et al.* [15] investigated the ethical implications of employing Artificial Intelligence (AI) and speech for earlier detection of AD. This emphasizes the significance of ethical considerations when implementing AI-powered AD detection systems. To diagnose AD, the research technique evaluates the potential risks and benefits of integrating vocal data with AI algorithms. These findings emphasize the necessity of reducing bias in AI models, acquiring informed consent, and safeguarding patient confidentiality. This study emphasizes the importance of incorporating ethical standards and protocols into the development and application of AI-driven technology for the early diagnosis of Alzheimer's disease. The primary goal was to ensure the independence and well-being of the patients.

Lu *et al.* [16] proposed a novel method for the identification of AD that employs the Salp Swarm Algorithm (SSA) to optimize fuzzy K-nearest neighbors. The significance of this study is its focus on improving the accuracy of AD diagnosis through the application of machine-learning techniques. The SSA optimization technique was combined with fuzzy K-nearest neighbors in the methodology, which led to an improved classification performance for the diagnosis of AD. The results of this study highlight the potential of the proposed method to surpass conventional methodologies, thereby enhancing the precision of early AD detection.

Ganesh *et al.* [17] investigated the application of CNNs in the detection of AD. The significance of this study lies in the application of CNNs, a potent DL tool, to enhance the accuracy of AD identification. To identify patterns indicative of AD pathology, the procedure involves instructing CNN models with neuroimaging data such as MRI or PET scans. These results emphasize the ability of CNNs to effectively distinguish between individuals with AD and healthy individuals, thereby providing a promising opportunity for early detection and intervention in AD.

AD is characterized by the accumulation of Amyloid-beta (Aβ) peptides, particularly detrimental oligomers (Aβo), as per Wang *et al.* [18]. The intricate nature and limited abundance of Aβo make accurate detection difficult. An innovative electrochemical sensing system employing gold nanostars (AuS) and a specific peptide probe (PrPc) provides exceptional sensitivity and selectivity for Aβo detection. Electrochemical Impedance Spectroscopy (EIS) was performed to investigate the binding of Aβo to PrPc-AuS. Significant changes in impedance were observed, enabling precise detection within a range of 5-200 pM and a minimal detectable concentration of 2 pM. This method has the potential to facilitate point-of-care diagnostics for neurodegenerative diseases and for early detection and monitoring of AD.

Helaly *et al.* [19] employed CNNs to identify and categories various stages of AD through the use of medical imagery. Two methodologies were implemented: transfer learning with VGG19 and fundamental CNN structures. In response to the COVID-19 standard, the proposed framework includes a web application specifically designed for remote authentication and authorization verification. The performance evaluation yielded favorable results, as evidenced by the nine criteria. The accuracies of the 2D and 3D multiclass AD stage classifications for the CNN architectures were 93.61% and 95.17%, respectively. Conversely, the fine-tuned VGG19 model achieved an accuracy of 97% in the classification of multiclass AD stages.

Arafa *et al.* [20] conducted an investigation that investigated the most recent developments in DL techniques for early identification and categorization of AD. It includes various stages of the procedure, including preprocessing, learning, classification, and imaging. The modalities examined included structural and functional MRI, as well as PET imaging, for the evaluation of brain metabolism and amyloid. The quality of the images was improved by emphasizing the preprocessing procedures. The popular DL algorithms employed for categorization were also examined. Despite the remarkable results, the challenges associated with categorization and preprocessing were resolved. This study examined the methodologies and results of numerous publications to provide a current assessment of the state of research on the detection and classification of AD.

Rafii *et al.* [21] investigated the present state of our understanding of AD at the preclinical stage, as well as the obstacles associated with conducting trials for this demographic. The emphasis is on advancements in plasma biomarker assays, recruitment strategies, cognitive assessment methods, and self-reported outcomes that have enabled the successful conclusion of phase 3 trials for preclinical Alzheimer's disease. This study also investigates the growing interest in anti-amyloid immunotherapy trials and recommends a systematic screening approach for identifying amyloid accumulation in clinically healthy individuals. The aim was to develop a therapy that is efficacious and can either prevent or delay cognitive decline.

In a multicenter case-control study, Cheung *et al.* [22] created a DL system that employed retinal images as a simple and noninvasive screening method for the diagnosis of AD and dementia. The system was trained, verified, and evaluated using data from 11 studies. It demonstrated a high level of accuracy (83.6%), sensitivity (93.2%), and specificity (82.0%) for the identification of AD and dementia. Furthermore, it achieved an AUROC of 0.93. Additional experiments with various datasets yielded accuracy rates that varied from 79.6% to 92.1% as well as AUROC values that ranged from 0.73 to 0.91. The system successfully distinguished between participants with amyloid-β-positive and amyloid-β-negative PET datasets. Subgroup analysis demonstrated that patients with both diabetes and eye disease demonstrated superior performance. This study underscores the potential of retinal images as a screening tool for Alzheimer's disease, as evidenced by the positive results obtained from the DL model.

Kong *et al.* [23] suggested a method for combining MRI and PET scans of individuals with AD to generate a fused image. The efficacy of the fusion strategy in dichotomous and multiclassification tasks was evaluated using 3D convolutional neural networks. The multimodal features of the combined images are enhanced by 3D convolution, which extracts the feature information. The extracted features are subsequently classified and predicted using a neural network with complete interconnections. The experimental trial using the AD Neuroimaging Initiative (ADNI) dataset demonstrated that the method outperforms previous methods in terms of specificity, sensitivity, and accuracy.

Gao, Shuangshuang [24], investigates the potential of DL to identify AD and emphasizes its advantages over traditional ML methods. The text concentrates on biomarkers associated with AD and the methods for extracting features. The application of DL models for AD detection was also examined. This study examined and combined a variety of AD detection methods and models, thereby illustrating the exceptional efficacy of DL technology in the detection of AD.

Helaly *et al.* [25] introduced DL-AHS, a DL framework that is specifically designed for the automatic segmentation of the left and right hippocampus in the diagnosis of AD. “The framework was trained using MRI data from the AD Neuroimaging Initiative (ADNI) and Neuroimaging Tools and Resources Collaboratory (NITRIC) datasets. It is further processed using Medical Image Processing, Analysis, and Visualization (MIPAV) and employs the U-Net architecture. Furthermore, it was improved using a Deep Convolutional Generative Adversarial Network (DC-GAN). The authors suggested two segmentation architectures: Simple Hyperparameter Tuning in U-Net (SHPT-Net), which entails the adjustment of hyperparameters, and RESU-Net, which employs transfer learning with ResNet blocks [18].” The SHPT-Net achieved an accuracy of 94.34% and a Dice similarity coefficient of 93.5%, while the U-Net with ResNet blocks (RESU-Net) achieved an accuracy of 97% and a Dice similarity value of 94%.

Orouskhani *et al.* [26] utilized DL techniques to overcome the constraint of having a limited number of image samples by utilizing few-shot learning. This approach was specifically designed to address the challenge of AD diagnosis. A novel deep triplet network employing the VGG16 architecture was introduced to analyze brain MRI data and identify AD. The network implements a conditional loss function to improve the model accuracy. The proposed model outperformed the existing models in terms of accuracy, as evidenced by the experimental results on the OASIS dataset.

Venugopalan, Janani, *et al.* [27] utilized DL techniques to assess a variety of data, including MRI scans, SNPs (single nucleotide polymorphisms), and clinical test results. They categorized individuals into three groups: control, MCI, and AD. Stacked denoising autoencoders are employed to extract features from clinical and genetic data, whereas 3D-CNNs are specifically optimized for the processing of imaging data. The high-performance characteristics acquired by the deep models were revealed using an innovative data analysis technique. It was observed that deep models perform better than superficial models, such as SVM and decision trees, based on the ADNI dataset. The accuracy, precision, recall, and mean F1 scores of the classification were enhanced by the integration of the multimodal data. In accordance with the most recent research on AD, the Rey Auditory Verbal Learning Test (RAVLT) and the hippocampus and amygdala brain regions have been identified as critical factors.

Sharma *et al.* [28] investigated computer-assisted diagnostic techniques intended to detect Alzheimer's disease. Three components were employed: sagittal, for analysis of the corpus callosum; frontal, for extraction of the hippocampus; and cortical variation. Support Vector Machine (SVM) classification was implemented. The proposed framework had a precision rate of 91.67% in identifying AD during its initial phases.

Ebrahimi *et al.* [29] addressed the challenge of early detection of AD using AI. Temporal correlations in three-dimensional (3D) MRI volumes were captured using sequence-based models in their approach. “To extract features, ResNet-18, a form of Convolutional Neural Network (CNN), was implemented. Conversely, sequence-based models, including Temporal Convolutional Networks (TCN) and recurrent neural networks, have been employed for sequence modelling. The Temporal Convolutional Network (TCN) model demonstrates the best classification performance, with a specificity of 92%, a sensitivity of 91.56%, and an accuracy of 91.78% [22].” The results suggest that the classification accuracy of 2D and 3D CNNs for AD detection was improved by a maximum of 10% when sequence-based models were employed.

Al-Shoukry *et al.* [30] emphasized the necessity of accurately diagnosing AD, particularly in its initial stages, to facilitate timely intervention and preventive therapies. This statement emphasizes the limitations of traditional machine detection methods while simultaneously emphasizing the potential of DL techniques for the early detection of AD. This study offers a succinct assessment of the relevant literature and investigates the potential of DL to assist researchers in the early diagnosis of Alzheimer's disease. Nevertheless, they are devoid of precise methodologies or definitive results.

Safi *et al.* [31] improved the accuracy of early detection of AD through the use of EEG data. The Hjorth criteria were implemented in addition to other frequently observed characteristics. The efficacy of various signal decomposition techniques, such as Empirical Mode Decomposition (EMD), Discrete Wavelet Transforms (DWT), and filtering into specific brain frequency ranges, was evaluated. Furthermore, the efficacy of classification algorithms, including SVM, KNN, and Regularized Linear Discriminant Analysis (RLDA), was assessed. By merging the Hjorth parameters with common features, the detection accuracy can be improved by preprocessing and gathering characteristics from the EEG signals of both healthy individuals and patients with Alzheimer's disease. By utilizing the KNN algorithm for classification and the Discrete Wavelet Transform (DWT) technique for signal decomposition, the highest level of accuracy achieved was 97.64% [31].”

A novel deep separable CNN model for AD classification was presented in a study conducted by Liu *et al.* [32]. This model effectively mitigates the deficiencies of contemporary machine learning algorithms, including the necessity for substantial training datasets and expensive hardware. The model is appropriate for mobile embedded systems because it utilizes Depth-wise Separable Convolution (DSC) to reduce the number of parameters and computing expenses. The experimental results of the OASIS MRI dataset indicate that AD can be effectively identified, and the performance of the model can be enhanced through transfer learning. The proposed model achieved average classification accuracies of 91.40% and 93.02% by utilizing sophisticated neural networks, such as AlexNet and GoogLeNet, respectively, while consuming minimal power. Table **1** illustrates State-of-the-Art Techniques for Early Detection and Classification of AD.

Table **1** illustrates the robust landscape of research and development efforts aimed at improving the early detection and classification of AD through a variety of innovative DL models, imaging techniques, and biomarker analyses. These advancements promise to revolutionize AD diagnosis by offering earlier and more accurate detection, which is crucial for effective intervention and management.

## 5 PARKINSON’S DISEASE

The studies summarized in this section highlight a diverse array of innovative approaches for diagnosing PD. These methods encompass advanced techniques for ML, DL, and biomarker detection, demonstrating significant improvements in accuracy and noninvasive diagnostics. By leveraging voice recordings and handwriting analyses to employ sophisticated neural networks and novel biomarkers, these advancements offer promising new avenues for the early detection and effective management of PD. Figure (**3**) illustrates the PET scans of the healthy and Parkinson’s brains.

Ali, Liaqat, and other researchers [33] propose a diagnostic method for detecting Parkinson's illness. The method involves two steps: refining the characteristics using a regularized linear SVM, followed by using a DNN for classification. The text highlights the importance of appropriate validation methods and demonstrates the effectiveness of the proposed system by utilizing voice recording-based datasets. The system achieved 100% accuracy in leave-one-out cross-validation and 97.5% accuracy in k-fold cross-validation, providing evidence of its usefulness. These findings surpass those of earlier methods, indicating the possibility of improved noninvasive assistance in diagnosing PD.

Islam *et al.* [6] investigated the use of ML and DL techniques to identify PD, focusing on analyzing speech, handwriting, and wave spiral datasets. The system evaluates multiple ML and DL techniques, including classifiers, to enhance the precision of diagnoses and support clinical decision-making. In addition, it explores the process of biomarker identification using various approaches, offering valuable insights for improving the diagnostic process. This review offers guidance for future research, outlining advancements in ML and DL techniques for the diagnosis of Parkinson's. This will benefit both the scientific community and medical practitioners.

In this study, Cuk *et al.* [34] examined the possibility of using LSTM with attention processes to detect PD by analyzing data from a dual-task walking test. The proposed approach utilizes an enhanced variant of the Crayfish Optimization Algorithm (COA) to enhance network efficiency. The proposed approach was evaluated using a real-world clinical gait dataset, and the most successful model achieved an accuracy of 87.4187%.

Veetil *et al.* [35] created a language-agnostic model for classifying PD using Variational Mode Decomposition (VMD) on sustained phonations in Spanish and Italian. A DL approach was employed to establish cross-lingual validity and to evaluate gender bias and robustness using authentic data. The results showed encouraging levels of cross-lingual accuracy, ranging from 65% to 80%. Within the same dataset, the accuracy was notably high, ranging from 90 to 95%. Furthermore, the model exhibited strong generalizability, achieving up to 63% accuracy when applied to an independent dataset. The proposed method demonstrated consistent performance across datasets that included multiple languages and the same linguistic demographics. It also performs well under various recording situations, indicating its potential for the accurate and widely applicable identification of PD from speech.

Chen, Qian, *et al.* [36] present a novel method for effectively identifying α-synuclein (α-syn) in the bloodstream, which is crucial for early detection of PD. The use of magnesium-based micromotors combined with Fe3O4/GOx nanoparticles and anti-α-syn antibodies significantly enhanced the detection of α-syn. The asymmetrical arrangement of the micromotors facilitated a more effective capture of α-syn in the circulation. A novel electrochemical detection device was developed to specifically identify α-syn and amplify its signal by harnessing the GOx catalytic activity. This innovative approach has the capacity to identify PD at an early stage and provide effective treatment.

Sun *et al.* [37] explored the application of fluorescence imaging techniques to investigate the pathophysiology of PD using bioactive chemicals. This text classifies and condenses fluorescent probes that specifically target biomarkers associated with PD, along with their use in the study of PD pathology. The purpose of this review is to offer valuable information for the development of fluorescence probes and to contribute to the study of the underlying causes and therapy of PD.

Cantürk *et al.* [38] proposed an AI approach to identify PD using voice signals. “The system employs scalogram images generated using a Continuous Wavelet Transform and evaluates them using various DL techniques. First, classifiers such as AlexNet, GoogleNet, ResNet50, and a hybrid system are used. Furthermore, an in-depth analysis was conducted on a feature fusion technique that utilizes DenseNet and NasNet. The deep feature fusion system attained 95% accuracy, representing a significant improvement of 38% compared with the ablation study. This study offers valuable insights into the application of DL models and feature fusion in the detection of Parkinson's [38].”

Rajinikanth *et al.* [39] suggested a strategy that utilizes Pre-trained Lightweight DL (PLDL) methods to precisely diagnose PD using hand sketching. The procedure includes image preprocessing, data augmentation, two-fold training, deep feature selection with dropout, and binary classification. This study achieved a perfect detection accuracy of 100% by utilizing the KNN classifier with the selected database.

Bakkialakshmi *et al.* [40] investigated the efficacy of DL methods for the early diagnosis and classification of PD. They utilized a dataset consisting of speech signal features from 3000 individuals diagnosed with PD. According to them, “Five deep learning models, namely ResNet50, VGG16, Inception v2, AlexNet, and VGG19, were trained and evaluated. The results demonstrated highly encouraging accuracies, ranging from 89% to 95%. AlexNet achieved the highest accuracy of 95% [40].” This work emphasizes the potential of DL in enhancing the diagnosis of PD and highlights the significance of utilizing aspects of speech signals for noninvasive evaluation.

Höglinger, Günter *et al.* [41] SynNeurGe is a classification approach for PD that is based on biological factors. It considers the presence of abnormal α-synuclein, evidence of neurodegeneration, and mutations in the pathogenic genes. The clinical components were coupled with these components for diagnosis. The objective is to bring fundamental and clinical research closer to precision medicine and treatments that can modify diseases. This concept highlights the importance of conducting prior validation while simultaneously recognizing ethical considerations and constraints.

The study conducted by Govindu *et al.* [42] examined the application of ML algorithms in telemedicine for the prompt detection of PD. Four ML models—SVM, RF, KNN, and LR—were developed and evaluated using MDVP audio data from a sample of 30 individuals, consisting of both PD patients and healthy individuals. The Random Forest classifier was the most efficient ML method, achieving a detection accuracy of 91.83% and a sensitivity of 0.95. This study proposes the use of ML in telemedicine to enhance PD diagnosis and improve patient outcomes.

The study conducted by Gupta *et al.* [3] examined the use of AI and ML in the diagnosis, treatment, and identification of PD biomarkers. The primary focus of this research was the utilization of artificial intelligence and ML techniques to detect Parkinson's disease. This is achieved by analyzing several characteristics, including neuroimaging data, speech recordings, and gait problems. The study also explored the potential applications of AI and ML in managing PD by examining alterations in lipidomics and the gut-brain axis. This study also explored the utilization of AI and ML algorithms to identify the early stages of PD. Additionally, it examines the integration of the metaverse, Internet of Things, and electronic health records to enhance the management of PD. Finally, it encompasses the utilization of AI and ML algorithms in neurosurgical procedures and drug development.

Sayed *et al.* [43] explored the potential of modifying voice features to predict PD onset. Advanced machine-learning techniques, including XGBoost, LightGBM, Bagging, AdaBoost, and SVM, were utilized in this study. “This study evaluated the prediction accuracy of various models using parameters such as accuracy, area under the curve (AUC), sensitivity, and specificity. The LightGBM model exhibited exceptional performance, achieving a remarkable accuracy rate of 96%, a corresponding AUC of 96%, a perfect sensitivity of 100%, and a specificity of 94.43%. This study highlights the significance of utilizing voice biomarkers and advanced machine-learning algorithms for the precise and timely detection of Parkinson's [43].”

Concha-Marambio, Luis, *et al.* [44] assessed the precision of the αS-Seed Amplification Assay (αS-SAA) in identifying misfolded α-synuclein aggregates in Cerebrospinal Fluid (CSF) as a biomarker for synucleinopathies such as PD. “An efficient αS-SAA method was employed to examine Cerebrospinal Fluid (CSF) samples from 206 individuals, including newly diagnosed PD patients, healthy individuals, and individuals with isolated Rapid Eye Movement Sleep Behavior Disorder (iRBD). The results demonstrated a 98% accuracy in identifying αSyn-seeds in PD-CSF and a 93% identification rate in iRBD patients. The assay demonstrated greater agreement with the final diagnosis than with the original diagnosis, suggesting its effectiveness for early disease detection. These results suggest that αSyn-seeds are detectable during the initial prodromal stage of synucleinopathies and serve as valuable indicators of the risk of synuclein-related diseases [44].”

Alalayah *et al.* [45] presented techniques for the early detection of PD using auditory signals. “This method uses Recursive Feature Elimination (RFE) to choose pertinent features and utilizes t-distributed Stochastic Neighbor Embedding (t-SNE) and Principal Component Analysis (PCA) to decrease the number of dimensions. The features were evaluated using five distinct classifiers: Support Vector Machine (SVM), K-Nearest Neighbors (KNN), Decision Tree (DT), Random Forest (RF), and Multilayer Perceptron (MLP). The findings indicate that the combination of RF with t-SNE produces an accuracy of 97%, whereas MLP with PCA obtains a higher accuracy of 98%, surpassing the results of earlier investigations [45].”

The study conducted by Wu *et al.* [46] examined the use of AI-based gait assessment for diagnosing and treating PD. This study conducted an extensive literature review to identify research publications on the use of AI to evaluate gait in individuals with Parkinson's. These results suggest that utilizing artificial intelligence to evaluate gait has the potential to decrease freeze episodes, improve diagnosis, and increase motor independence in individuals with PD. These technologies offer improved diagnostic precision, ongoing monitoring, and customized therapeutic interventions, which have the potential to change clinical decision-making and provide information for tailored treatments. Additional investigations are necessary to ascertain the effectiveness of these methods and improve their implementations.

Wang *et al.* [47] presented a comprehensive analysis of the aggregation of α-synuclein (α-Syn) in PD and investigated various methods for its detection both in living organisms and in laboratory settings. These approaches include mass spectrometry, antigen-antibody detection, electrochemical sensors, and imaging. Early detection of α-syn aggregates in Parkinson's can aid in disease intervention and monitoring disease progression.

Mahmood *et al.* [48] developed a practical model for the early detection of PD through the extraction of crucial vocal characteristics. Current diagnostic methods have limitations that highlight the need for novel models. The proposed model exhibited an RMSE error of 0.10, which was superior to that of the current models. Moreover, it enables practitioners to consistently monitor patients by using the Total Unified PD Scale. This procedure involves collecting the essential vocal components that are crucial for the early detection of illnesses.

Parajuli *et al.* [49] introduced advanced techniques based on DL to identify MCI in the sleep Electroencephalograms (EEGs) of patients with Parkinson's disease. “The procedure involves dividing the EEG time series into different sleep stages, processing data using continuous wavelet transform and variational mode decomposition, and employing convolutional neural networks. The variational mode decomposition-based model has superior performance in terms of accuracy, sensitivity, specificity, area under the curve, and quadratic weighted kappa score (all exceeding 99%) when compared to the continuous wavelet transform-based model. Changes in middle- and high-frequency variational mode decomposition components across sleep stages were used to identify MCI-related traits in Parkinson's disease. This method offers a promising computer-aided diagnostic tool for identifying MCI and monitoring the progression of PD [49].”

Abdullah *et al.* [50] proposed a highly effective DL model that can accurately detect PD at an early stage using handwritten records. The model employs a genetic algorithm to optimize the features and utilizes the KNN method for classification. The findings provide a detection accuracy exceeding 95%, a precision of 98%, an area under the curve of 0.90, and a slight loss of 0.12. The detection capability was significantly improved compared with that of earlier methods.

Yang, Mingjing, *et al.* [51] developed a PD identification algorithm that is easy to understand, using whole-brain MRI data. A 3D ResNet model was developed to classify PD, with average accuracies of 96.1% for cross-validation and 94.5% for held-out datasets. In addition, the 3D Gradient-weighted Class Activation Mapping (Grad-CAM) technique was employed to visualize brain regions associated with Parkinson's. Notable discrepancies were observed in the frontal lobe, which were associated with linguistic semantic difficulties and Unified PD Rating Scale (UPDRS) scores. This study provides evidence for the involvement of the frontal lobe in the development of Parkinson's.

Zhang *et al.* [52] present a novel approach to improve the detection of PD using voice data that relies on Fractional Attribute Topology (FrAT). This procedure involves utilizing a fractional Fourier transform on speech samples to produce spectrograms of different orders. Subsequently, the energy variation information was quantified and used to construct FrAT. The associated component characteristics representing the structural properties of FrAT were subsequently extracted and used for classification. The proposed approach achieved classification accuracies of 99.57, 95.33, and 94.13% on three datasets comprising different native languages. This illustrates the effectiveness of the approach in identifying PD and the significance of attribute topology in this field.

Li, Kuan *et al.* [53] developed two hybrid DNNs that enhance the accuracy of classifying PD by utilizing EEG inputs. These models combine convolutional neural networks with long short-term memory to form hybrid architectures that incorporate both parallel and sequential processing. Deep-CNN extracts structural information from Electroencephalogram (EEG) signals and subsequently transmits it through an LSTM network to identify context dependency. The parallel model demonstrated a specificity of 97.6%, a sensitivity of 97.1%, and an accuracy of 98.6%. In contrast, the series model achieved a specificity of 99.1%, sensitivity of 98.5%, and accuracy of 99.7% when classifying three groups: PD patients with medication, PD patients without medication, and healthy individuals.

Shcherbak *et al.* [54] proposed the use of wearable sensors and ML to differentiate between those who are in good condition and those who have stages 1 and 2 PD. Using publicly available sensors, data were collected from 113 people who performed 11 popular workouts. ML techniques were employed to extract features, reduce dimensionality, and classify data to enhance the accuracy of PD diagnosis in its first phases. The results demonstrate encouraging F1-micro scores of 0.78 and 0.88 for PD stages 1 and 2, respectively, suggesting that the proposed technique is effective in identifying early PD without invasive treatments.

Ali, Liaqat, and other researchers [55] developed a distinctive ensemble method for predicting PD based on voice data. To enhance precision and broaden applicability, this methodology integrates feature selection with Deep Neural Networks (DNNs). In addition, the ensemble model EOFSC incorporates the latest findings on the optimal models for different speech data formats and sample relationships. EOFSC employs many base classifiers that are responsive to different subsets of features, and combines them to make a final prediction using a popular vote approach. Research findings indicate a 6.5% improvement in the accuracy of PD identification compared with traditional methods. The proposed approach surpasses conventional Deep Neural Networks (DNNs) and other integrated systems, demonstrating the efficacy of incorporating feature selection with DNN in predicting PD.

Zhao *et al.* [56] proposed a novel hybrid method for detecting PD by analyzing handwriting. The model employs a three-layer CNN and a bidirectional gated recurrent unit to extract pertinent features from three handwriting assessments (meander, circle, and spiral). The proposed model surpassed several state-of-the-art methods, achieving identification rates of 92.91%, 85.71%, and 90.55% in the three corresponding tests. This technique effectively utilizes sequential handwriting data to detect indications of Parkinson's, making it a practical tool for noninvasive diagnosis and monitoring of the condition.

The study by Gupta *et al.* [3] explored the application of AI and ML in the management of PD, focusing specifically on diagnosis, treatment, and biomarker discovery. This study examined their use across several modalities, such as neuroimaging, voice analysis, and gait evaluation. We also explored the potential of AI to treat PD by altering lipidomics and manipulating the gut-brain axis. This review highlights the importance of the early detection of PD using AI and ML methods, which are supported by emerging technologies such as the metaverse and IoT. Additionally, it examined their roles in neurosurgical procedures and drug development, highlighting their importance in patient treatment and therapeutic strategies. In summary, AI and ML offer advantages in enhancing the management of PD through early detection, optimizing treatment approaches, and enhancing overall patient outcomes.

Hireš, Máté *et al.* [57] presented a collection of CNNs designed to identify PD from voice recordings accurately. The methodology narrows the gap between pre-training and target tasks by fine-tuning the underlying CNN with multiple techniques. The methodology was evaluated using voice recordings of individuals diagnosed with Parkinson's and a control group without the condition. The outcomes demonstrated favorable results for all frequencies. The vowel/a/had superior performance, with 99% accuracy, 86.2% sensitivity, 93.3% specificity, and an AUC of 89.6%. The results indicate that the proposed method can be employed for screening, diagnosing, and monitoring Parkinson's disease. Additionally, it has the advantage of online voice recordings based on vowel sounds without the need for additional equipment. Table **2** illustrates state-of-the-art methods for PD diagnosis and management.

Table **2** presents a comprehensive overview of the latest methods and technologies used for PD diagnosis and management. These state-of-the-art approaches encompass a range of techniques, including ML, DL, and advanced imaging, and have demonstrated significant advancements in accuracy and efficiency. Ongoing research in this field highlights the potential of these innovative methods to improve early detection, treatment, and patient outcomes in Parkinson's disease.

## 6 EPILEPSY

Several recent studies have made significant advances in the field of epilepsy detection using innovative methodologies and applications of ML and DL. Abdukodirov *et al.* [58] investigated the potential of hyperdimensional computation for the detection of epilepsy using wearable devices in their study. This method has advantages in terms of minimal memory requirements and simplicity. This study illustrates innovative methods for the examination, evaluation, and enhancement of ML models for the detection of epilepsy that are not feasible using conventional methodologies. The efficacy of epilepsy detection is enhanced by evaluating the similarity between individual models, generating general models from them, and merging personal and general models to create hybrid models. The transfer of information between models trained on various datasets was analyzed, resulting in valuable insights for the disciplines of neuroscience and engineering. Figure (**4**) illustrates an abnormal Brain MRI in Epilepsy.

An advanced method for predicting epilepsy was presented by Pale *et al.* [59] based on EEG signal analysis. The objective was to resolve the obstacles encountered in the previous methodologies. This technology employs a Bidirectional LSTM (BiLSTM) network in conjunction with a multi-head self-awareness system to accurately identify patients across multiple locations. The scalp EEG data analysis of the CHB-MIT database produced extraordinary results, including a sensitivity of 96.5%, specificity of 97.04%, F1-score of 96.6%, and accuracy of 96.2%. The data indicate that the proposed technique is capable of reliably identifying seizures across multiple individuals as well as demonstrating a superior ability to recognize epilepsy patterns and maintain stability.

Additionally, Dutta *et al.* [60] introduced a novel method for diagnosing epilepsy that utilized dynamic EEG channel screening. This method improves the reliability of the detection and enhances the extraction of pertinent characteristics. The complexity and dynamic screening of EEG methods were assessed using fine composite Multiscale Dispersion Entropy (RCMDE). Subsequently, it identifies common epileptic characteristics across various participants within the 3-15 Hz frequency range. A Residual Convolutional Long Short-Term Memory (ResCon-LSTM) neural network is developed to detect epilepsy in a variety of individuals. The results of experiments conducted on the CHB-MIT dataset are exceptional: a single-subject experiment achieved a detection accuracy of 98.523%, a remarkable 5.298% improvement over non-channel screening. The ability of the approach to sustain exceptional detection performance across a broad spectrum of participants was substantiated by an average accuracy of 96.596% in the across-subject trial.

Single-channel EEG recordings are examined for epilepsy using a classification methodology developed by Li, Yang, and their collaborators [61]. They utilized a dynamic graph neural network with an attention mechanism. The procedure involves optimization of the adjacency matrix and generation of graphs through Empirical Mode Decomposition (EMD). A multilayer dynamic graph neural network with an attention mechanism was employed to acquire knowledge regarding the biased graph characteristics, and feature integration was performed using an MLP-pooling structure. The results of the experiments conducted at the University of Bonn’s epileptic EEG database were favorable. An overall score of 99.47% was obtained across 12 classification tasks, with specificity of 99.91%, sensitivity of 99.78%, and precision of 99.87%, resulting in an average accuracy of 99.83%. These results were achieved by applying 25 cycles of ten-fold cross-validation.

In this investigation, Komal *et al.* [62] assessed seizure detection systems capable of remotely monitoring individuals with epilepsy. The evaluation focused on the technical specifications, performance, user preferences, and efficacy of these systems. In accordance with the PRISMA guidelines, the analysis was conducted on 30 of the initial 1095 documents. A total of 16 noninvasive devices were identified, each equipped with advanced sensors capable of measuring EEG, muscle activity, and motion detection using accelerometers. Skin irritation, false alarms, discomfort, and diminished battery life are among these issues. These devices were not utilized by 3.2 out of 5 individuals with epilepsy due to their cost and concerns regarding their appearance, as indicated by the literature. Effective collaboration among physicians, technology professionals, and researchers is essential for the advancement of device development, as it is centered on fulfilling user expectations and requirements.

Dash *et al.* [63] investigated various ML and DL techniques for the identification of epileptic seizures using EEG data. This study examines the efficacy of various methodologies, including SVM, ANN, CNN, and LSTM, when applied to time-domain, frequency-domain, and time-frequency domain characteristics. Furthermore, it investigated the utilization of alternative physiological indicators, including accelerometer and Electrocardiogram (ECG) data. The proposed LSTM-based method for classifying seizure-nonseizure EEG data achieved an accuracy of 96.5%. This report offers a comprehensive overview of current approaches and provides recommendations for future research, such as the investigation of sentiment analysis as a viable approach for seizure identification.

A novel method for the effective identification of Epileptic Seizures (ES) through the analysis of EEG signals was presented in the study conducted by Ahmad *et al.* [64]. “The methodology entails the analysis of EEG data, identification of appropriate characteristics through coefficient and distance correlation analysis, and implementation of a Bagged Tree-based classifier (BTBC). Compared to prior ML models, the recommended method exhibits superior performance, with an average accuracy that is 2% higher on the Bonn and UCI-EEG benchmark datasets. The decision-making processes of the framework were investigated using the SHapley Additive Explanation (SHAP) method [64].” In general, this technique yields positive results by accurately categorizing ES and improving the diagnostic process for individuals with neurological disorders.

Chari *et al*. [65] employed a lesion-detection technique to identify Focal Cortical Dysplasia (FCD) in children who were undergoing Stereo Electroencephalography (SEEG) for the evaluation of drug-resistant epilepsy in a forthcoming investigation. Structural MRI sequences were analyzed using an approach to identify clusters of Focal Cortical Dysplasia (FCD) following SEEG planning. Three additional SEEG electrodes were inserted if the three highest-ranked clusters were not included in the sample. One of the 20 individuals in the group received additional electrode connections in the Seizure-Onset Zone (SOZ), while the other three had clusters placed in the SOZ. The median patient age was 12 years. With no complications, nine individuals underwent implantation of an additional 16 electrodes. The algorithm's initial validation for the diagnosis of the Seizure Onset Zone (SOZ) in children undergoing Stereo Electroencephalography (SEEG) is demonstrated in this study, underscoring the importance of a comprehensive prospective evaluation before its practical implementation.

Anita *et al.* [66] introduced a sophisticated DL framework. “The objective of this architecture is to autonomously identify epileptic seizures through analysis of EEG data. Traditional datasets have been used for the acquisition of EEG signals. The Fourier-Bessel Series Expansion-Based Empirical Wavelet transform (FBSE-EWT) method was employed to decompose the signals, and an autoencoder was employed to extract features. The Relief-F feature ranking method was implemented to minimize computational resources and select significant features. For classification purposes, a Hybrid Deep Scheme (HDS) combines Multi-Scale Atrous-based Deep CNN (MSA-DCNNs) with LSTM. ASMBWO optimizes the parameters. The proposed method surpassed the current techniques, as evidenced by experimental data [66].”

Pouryosef *et al.* [67] introduced a novel approach for the precise detection of EEG signals in Brain-Computer Interface (BCI) applications. The pipeline employs bat and genetic algorithms to generate features and reduce dimensionality. The Bat technique was implemented to identify and classify the characteristics subsequent to the separation and categorization of harmonics. In place of k-nearest neighbors and naïve Bayes, genetic algorithms and neural networks have been implemented to recognize EEG signal segments. The dataset includes signals from healthy individuals with either open or closed eyes, as well as epileptic patients who participate in activities that either trigger or do not trigger seizures. Compared to previous methodologies, the results demonstrate higher precision and efficacy, with a minimum accuracy of 100% for balanced classes and 75.9% for unbalanced classes. The efficacy and precision of this method for the clinical analysis of epileptic EEG signals are promising.

Basha *et al.* [68] utilized the Bonn University dataset to identify epileptic seizures in EEG signals using ML and DL models. The study comprises data from 100 patients with 'S' (ictal) and 'Z' (normal) segments recorded at a frequency of 173.61 Hz, despite the dataset for DL being restricted. Variance, power, skewness, and kurtosis were analyzed to determine the epileptic characteristics. The CGRU-SVM model outperformed all other classifiers, achieving an accuracy rate of 97.54%. To extract unique attributes, illuminate the peculiarities of epileptic seizures, and demonstrate the capacity of DL models to identify them, statistical measurements are implemented. Diverse evaluation procedures and variable input sets should be the focus of future research.

Goel *et al.* [69] identified epileptic seizures by analyzing EEG signals. The software employs transfer learning techniques to identify features and generate recurrence graphs from EEG time series. PCA reduces computational burden by identifying and selecting the relevant features. A variety of classifiers, including decision trees, KNN, Gaussian, random forests, bagging, and SVM, were used to process the collected features. Compared to all other algorithms, the SVM algorithm exhibits superior performance, with a sensitivity of 98.15%, precision of 99.61%, and accuracy of 98.21%. This study examines the performance of classifiers with and without feature selection and concludes that feature selection improves the performance. This emphasizes the efficacy of deep-learning-based feature extraction. The proposed methodology effectively distinguishes between epileptic and non-epileptic EEG data and has the potential to aid in the prevention of seizures.

The Spiking Conformer, a neuromorphic model devised by Chen *et al.* [70], is intended to identify and forecast portions of epileptic episodes using extensive EEG recordings. The CHB-MIT EEG dataset was used to evaluate this model. In contrast to non-spiking models, the Spiking Conformer model reduces computational expenditure by employing spike-based addition operations. The processing required is significantly reduced, while maintaining a high level of accuracy, when a layer of spiking neurons is used in conjunction with an approximation technique. When unprocessed EEG data were employed, the Spiking Conformer achieved a specificity of 99.3% and a sensitivity of 94.9% for seizure detection. Furthermore, it had a specificity of 89.5% and a seizure prediction accuracy of 96.8%. The Spiking Conformer achieves exceptional results with approximately ten times fewer processes than the non-spiking models.

Tasci *et al.* [71] developed a unique method for automatic identification of epilepsy by analyzing a substantial EEG dataset that included 10,356 signals from 121 individuals. In addition to a framework based on hypercube patterns, a feature engineering model that extracts features from 35 channels was presented. A total of 245 feature vectors comprising statistical information and a Multilayer Discrete Wavelet Transform (MDWT) were generated by a hypercube-based feature extractor. Neighborhood Component Analysis (NCA) is a method that identifies critical features, which are subsequently incorporated into a k-Nearest-Neighbors (kNN) classifier through Leave-One-Subject-Out (LOSO) Cross-Validation (CV). The classifier achieved an accuracy of 87.78% in classification by voting and 79.07% using LOSO CV. This feature-engineering model, which employs fusion techniques, achieves exceptional performance in the identification of epilepsy when applied to the most comprehensive EEG dataset currently available.

Two distinct methods for distinguishing healthy, interictal, and ictal cases in epilepsy detection have been presented by Ilias *et al.* [72]. These methods eliminate the need for time-consuming feature extractions. Initially, the short-time Fourier Transform (STFT) was applied to the single-channel EEG data to generate images. These images were subsequently incorporated into pretrained models such as EfficientNet, DenseNet201, and AlexNet. In addition, this paper introduces a multimodal deep neural network that utilizes two branches of CNNs to extract features from single-channel EEG data. The STFT-based images were then loaded into a pretrained EfficientNet-B7 model. The significance of each modality is regulated by a gated multimodal unit. The University of Bonn's EEG database exhibits outcomes analogous to those of state-of-the-art methodologies through an examination of five distinct scenarios.

PredictMed-Epilepsy, a component of the PredictMed healthcare system, was first presented by Bertoncelli *et al.* [73]. It is intended to predict the occurrence of epilepsy in children with Cerebral Palsy and developmental disabilities. A longitudinal multicenter investigation involving 102 children examined factors such as the cause of Cerebral Palsy, type of epilepsy, and motor abilities. The PredictMed ML model identified correlations between epilepsy and factors such as scoliosis, communication impairment, Cerebral Palsy, and impaired motor function. The predictive model achieved an average accuracy, sensitivity, and specificity of 82%. Additionally, a Multi-Agent System (MAS), denoted PredictMed-Epilepsy, was created to facilitate the real-time detection of events through the patient Observing MAS. This research contributes to understanding the computational characteristics of at-risk young individuals and establishes a framework for the real-time monitoring of epilepsy therapy.

Epilepsy-Net, a collection of advanced ML methods for identifying epileptic seizures in EEG signals without the need for specialized feature extraction, was presented by Lebal *et al.* [74]. The Epilepsy-Net framework integrates RNNs, 1D-CNNs, and attention techniques, such as ResNet, Inception, gated recurrent units, and convolutional block attention modules. This study is the first to integrate the inception deep network technique with an attention mechanism to evaluate EEG signals for epilepsy identification. The model's accuracy was verified using major public EEG datasets, resulting in high accuracies of 100%, 99.05%, and 98.22% on the Bonn EEG dataset, the modified version of the Bonn EEG dataset, and the CHB-MIT dataset, respectively.

Farooq *et al.* [75] conducted a systematic literature analysis focusing on the performance of the classification and the method of feature selection in the automated diagnosis of epileptic episodes. This article analyzes the feature extraction techniques and classifiers that are frequently employed, utilizing data from reputable archives, such as MDPI and IEEE Xplore, to accomplish accurate classification of normal and epileptic seizures. To summarize state-of-the-art solutions, a classification system was developed, and benchmark datasets were evaluated for their intended purpose and lack of bias. The report concludes by identifying gaps, issues, and potential areas for future research in epilepsy prediction, and by offering a comprehensive analysis of the classifier functionality.

Majzoub *et al*. [76] employed ML, specifically the AlexNet Convolutional Neural Network (CNN), to analyze EEG inputs to identify epileptic seizure states. The accuracy of the predictions was investigated in this study by examining the impact of the selection of the training and test datasets. The results suggest that the training accuracy was 94.44% when samples from each patient were used and 92.98% when a subset was used. In both scenarios, binary categorization achieved a maximum accuracy of 98%.

The study conducted by Al-Hajjar *et al.* [77] examined the implementation of ML algorithms and the Internet of Medical Things (IoMT) in the identification of epilepsy. This finding emphasizes the significance of early seizure identification in the prevention of mortality. Remote monitoring and diagnosis of epilepsy are enabled by the incorporation of cloud services, ML techniques, and the Internet of Medical Things (IoMT). This combination enhances operational efficiency and decreases healthcare expenditure. This article provides a thorough analysis of sophisticated ML applications employed to diagnose epilepsy within the Internet of Medical Things (IoMT) framework. This underscores the substantial influence of these technologies on the healthcare sector.

EpilepsyNet, a DL transformer model, was developed by Lih *et al.* [78]. It is intended to identify and trace epilepsy by analyzing the EEG signals. The model employs the Pearson correlation coefficient to convert 5-second epochs into matrices, utilizing 1-minute segments from each participant. EpilepsyNet demonstrated exceptional performance in the classification of epileptic seizures, with a positive predictive value of 82%, sensitivity of 82%, specificity of 87%, and accuracy of 85%. These results were validated using ten-fold cross-validation and obtained through the integration of positional encoding. This method exhibits potential for neurological diagnostics that are both precise and effective, transcending the limitations of epilepsy detection.

Saminu *et al.* [79] examined the integration of artificial intelligence techniques, such as DL and ML, into Computer-Aided Devices (CAD) that are designed to detect epileptic seizures. This paper investigates the obstacles associated with the analysis of EEG signals and the potential of artificial intelligence methods to aid in the development of intelligent Internet of Medical Things (IoMT) devices. Although ML has been employed to detect seizures, subsequent studies have primarily focused on DL. This study emphasizes the potential of DL algorithms for improving mobile health solutions in clinical settings.

A real-time method for identifying epileptic seizures using EEG data was described by Shen *et al.* [80]. The tunable-Q wavelet transform and CNN were employed in this method. Statistical moments and spectral band power were employed to convert the EEG data into temporal and frequency domain information. Subsequently, these attributes were fed into a CNN. The CHB-MIT database was employed for evaluation, resulting in a remarkable accuracy rate of 97.57% and a sensitivity rate of 98.90%. Furthermore, it exhibits a latency of 10.46 seconds and a low false-positive rate of 2.13%. The proposed approach is highly promising for practical applications in the detection of seizures because of its ability to be implemented in real time in clinical settings.

Qiu *et al.* [81] introduced DARLNet, a hybrid DL model that combines ResNet and LSTM to detect epileptic seizures. The DARLNet model incorporates a channel attention module to highlight relevant data and a difference layer to extract supplementary seizure information, thereby addressing the deficiencies of the previous models. The Bonn Electroencephalogram dataset was employed to evaluate the performance of DARLNet in tasks that involved the identification of seizures in two and five categories.

A hybrid DL approach for the identification of epileptic episodes in EEG recordings is presented in a paper by Ahmad *et al*. [82]. The K-means Synthetic Minority Oversampling Technique (SMOTE) was employed to resolve the imbalanced data issue. This is subsequently combined with a 1D-CNN and Bidirectional LSTM network, utilizing Truncated Backpropagation Through Time (TBPTT) to precisely extract spatial and temporal sequence information. The proposed architecture utilizes sigmoid and softmax classifiers to classify seizures into binary and multiple categories. Compared with both baseline DL approaches and more recent approaches, the UCI epileptic seizure detection dataset exhibited superior performance in terms of precision, sensitivity, specificity, and F1-score when assessed using 10-fold cross-validation.

Miltiadous *et al.* [83] conducted a systematic review that examined various methods for the identification of epilepsy by analyzing EEG waves. Signal processing techniques, categorization procedures, and assessment databases were used to evaluate 190 papers. The review emphasises the growing utilization of Convolutional Neural Networks with Time-Frequency Decomposition Methodology for image analysis [83].

Ahmad *et al*. [84] investigated the most recent developments in DL and ML technology, which are based on Electroencephalography (EEG) for the purpose of identifying epileptic seizures. The text assesses a variety of methodologies for extracting statistical features and utilizing ML/DL models, highlighting their advantages, limitations, and challenges. Furthermore, it provides recommendations for the selection of the most effective feature extraction techniques and ML/DL models to improve seizure detection. These findings are intended to assist researchers in improving the efficacy of EEG-based epileptic seizure diagnosis.

Tawhid *et al.* [85] presented a framework based on ConvLSTM that is intended to diagnose epilepsy by analyzing EEG data. Prior to being submitted to the ConvLSTM model, the EEG data underwent segmentation and preprocessing. The efficacy of the model was evaluated by utilizing two datasets with varying population sizes. Five-fold cross-validation and Leave-One-Out Cross-Validation (LOOCV) techniques were employed to eliminate biases. The postulated paradigm was further supported by ablation investigations. The potential of the model as an automated system for diagnosing epilepsy is demonstrated by its superiority over current techniques.

Shoeibi *et al.* [86] offer a comprehensive explanation of DL algorithms that are employed to predict and identify epileptic episodes through the use of neuroimaging techniques. This article addresses DL models employed in this context, datasets, preparation procedures, and Computer-Aided Diagnosis Systems (CADS) based on DL. The research also investigates rehabilitation technologies, including hardware implementations of DL methods and Brain-Computer Interfaces (BCI). The discussion section examines the challenges and potential directions for future research in the field of epileptic seizure detection and prediction. This conclusion offers a succinct summary of the primary discoveries and insights presented in this research.

Sunaryono *et al.* [87] suggested a technique for autonomously identifying epilepsy from EEG signals using a combination of gradient boosting machines. “DFT and DWT were employed to preprocess the EEG signals, following which feature extraction was conducted. Prior to categorization, a genetic algorithm prioritized distinguishing attributes. The methodology was assessed using a dataset obtained from the University of Bonn, which was divided into three distinct categories: normal, interictal, and ictal. The experimental results indicate that the proposed GBM fusion method accurately identifies epilepsy with a 100% accuracy rate, thereby surpassing the performance of a single GBM. However, the model results may not be applicable to a wide range of EEG datasets [87].”

Tuncer *et al.* [88] suggested a method for the automatic identification of focal, generalized, and other epileptic seizures using EEG signals. This study compares conventional classification algorithms and DL methods for both two-class and four-class classification problems. The methodology consists of preprocessing scalp EEG data, extracting features using the discrete wavelet method, selecting features using Correlation-based Feature Selection (CFS), and classifying results using KNN, SVM, RF, and LSTM. A scalp EEG dataset from the Temple University Hospital (TUH) was used to assess the proposed approach. Employing an LSTM DL architecture, the method achieved a classification accuracy of 95.08% for the two-class problem and 95.92% for the four-class problem.

Shen *et al.* [89] offered a comprehensive description of a real-time electroencephalogram (EEG)-based method for identifying epileptic episodes. This method utilizes eight eigenvalue algorithms and a discrete wavelet transform to extract characteristics from numerous sub-frequency bands. Support vector machines are employed to classify data into three categories: health control, seizure-free, and seizure-active. The RUSBoosted tree ensemble method was subsequently implemented to facilitate the timely identification of seizure onset. Two datasets were employed for evaluation purposes: UB, which represents short-term data, and CHB-MIT, which represents long-term data. Both datasets are publicly accessible. In the three-class classification, the UB dataset exhibited a 97% accuracy and 96.67% sensitivity. Conversely, the CHB-MIT dataset demonstrated a 3.24% false-positive rate, 96.38% accuracy, and 96.15% sensitivity in real-time seizure detection.

Forooghifar *et al.* [90] developed a wearable system that is self-aware and specifically engineered to precisely detect epileptic seizures in real-time. The method utilizes a multiparametric ML technique to analyze cardiac and respiratory responses from ECG signals to identify seizures. The proposed system includes energy-quality trade-offs to improve battery longevity and self-awareness to enable extended monitoring. The significance of self-awareness was underscored by an examination of an epilepsy database from Lausanne University Hospital, which revealed a sensitivity of 88.66% and specificity of 85.65%. The battery life of the system is maintained between 67.55 and 136.91 days as a result of its self-awareness. Furthermore, it surpasses the specificity and sensitivity of the state-of-the-art techniques, which range from 85.54% to 79.33%.

To autonomously diagnose epilepsy from EEG signals, Jiwani *et al.* [91] introduced a CNN-LSTM model. This model accounts for both the temporal and spatial components of the signals. The model is well-suited for real-time processing owing to its exceptional specificity, sensitivity, and accuracy. It achieves a 100% accuracy, sensitivity, and specificity by accurately distinguishing between individuals in excellent health and those who experience seizures.

Beeraka *et al.* [92] utilized DL models and FPGA implementations of the Short-Time Fourier transform (STFT) block to improve the identification of epileptic seizures from EEG data. The methodology entails the use of CNN and Bidirectional-LSTM for seizure recognition, frequency band selection, feature extraction, and time-frequency analysis with Short-Time Fourier Transform (STFT). The Bonn EEG dataset was used for evaluation. A maximum discrepancy of approximately 0.13% was observed when comparing the STFT outputs derived from the hardware architecture and simulation results. The mean classification accuracies of the CNN and Bi-LSTM models were 93.9% and 97.2%, respectively, across all frequency bands for individuals with and without epilepsy.

Singh *et al.* [93] introduced a smart neurocare solution that incorporates cloud and fog computing to detect epileptic seizures in real time. This was accomplished by employing DL techniques on unprocessed EEG signals. It employs a channel-selection strategy based on maximal variance and utilizes single-channel EEG signals to improve computational efficiency. This method employs stacked autoencoder classifiers, RNN, and CNN to analyze the temporal structure of EEG segments. The simulation results indicate that the CNN-based strategy outperforms conventional methods, obtaining an accuracy of 96.43%, a sensitivity of 100%, and a specificity of 93.33% when applied to 30-second EEG segments from the CHB-MIT dataset. On the Bonn dataset, the CNN-based approach obtains 100% accuracy, sensitivity, and specificity for EEG segments that span 23.6 seconds. Seizures can be swiftly and precisely identified in environments with dense fog or clouds using this method.

A novel method for automated detection of epilepsy using Electroencephalography (EEG) data was devised by Sameer *et al.* [94]. By employing a 1D Convolutional Neural Network (CNN) to autonomously extract characteristics from EEG data, the proposed approach deviates from previous methodologies. These features were subsequently identified using conventional ML algorithms. This method achieves high accuracy while considerably reducing the duration of training compared with end-to-end DL models. The dataset was subjected to an experiment designed to evaluate its accuracy in distinguishing between healthy patients and those experiencing seizures. The highest accuracy level was 99.83%. It is well-suited for real-time clinical applications owing to its compact processing.

Barneih *et al.* [95] have developed an ANN model that analyzes EEG signals to identify epileptic seizures. The STFT technique was employed by the model to extract statistical features from the five EEG frequency bands. In various scenarios, performance measurements suggest that the accuracy rates can exceed 100%.

Table **3** provides an overview of recent advancements in epilepsy detection, highlighting the diversity of methods and datasets as well as the impressive accuracy and specificity achieved by these state-of-the-art techniques.

## 7 HUNTINGTON’S DISEASE

Recent advances in the research of HD have significantly deepened our understanding of its early symptoms, progression, and potential therapeutic strategies. Studies have examined cognitive impairments in gene carriers before symptom onset, explored promising treatments targeting mutant proteins, and identified biomarkers using advanced imaging and ML. These findings are pivotal for developing early diagnostic tools and effective interventions, offering hope for the improved management and treatment of HD. Figure (**5**) illustrates MRI scans of a Huntington’s disease (HD) sufferer’s brain (left) and a normal brain (right).

Paz-Rodríguez *et al.* [96] compared the cognitive abilities of individuals who possess the HD gene and those who do not, with a particular emphasis on the proximity of the two groups to the onset of symptoms. The HDC cohort comprised 41.1% of 146 participants. The disease burden and distance to the manifest stage of patients with HDC were assessed, and these variables were correlated with cognitive function. Compared to the non-HDC group, individuals in the HDC group who initially experienced symptoms demonstrated inferior performance on tests that assessed information processing speed and attention. This illustrates that patients with HD experience prefrontal cognitive impairment prior to the diagnosis of their motor symptoms.

The therapeutic potential of monoclonal antibody C6-17 was investigated in a study conducted by Bartl *et al.* [97]. In mice with HD, this antibody targets the mutant Huntingtin (mtHTT) protein. The antibody was administered to YAC128 HD rodents on a chronic basis; it was designed to target an exposed region adjacent to the aa586 Caspase 6 cleavage site. The antibody spread to both the periphery and the central nervous system, as evidenced by the reduced detection of HTT protein. In terms of body weight, motor behavior, and the rate of impairment progression, mice administered mAB C6-17 demonstrated superior performance compared to the control groups. These findings indicate that the antibody-based approach has significant potential as a treatment for HD.

At 3, 6, and 11 months of age, Nicholas Vidas-Guscic *et al.* [98] employed diffusion MRI to examine white matter abnormalities in zQ175DN Heterozygous (HET) mice models of HD. Conventional Diffusion Tensor Imaging (DTI) and advanced Fixel-Based Analysis (FBA) were implemented to detect abnormalities in particular regions of the brain. The corpus callosum genu experienced a significant reduction in fiber cross-section and density, as determined by FBA. The striatum exhibited the most significant anomalies in the initial alterations observed in HET animals using DTI and FBA measurements. These abnormalities were caused by increased quantities of neurofilament light chain proteins rather than myelin deficiency. The sensitivity of FBA metrics was greater than that of DTI metrics, indicating that they have the potential to serve as non-invasive biomarkers for the progression of HD and the assessment of treatment efficacy in both animal and human models.

This cross-sectional study investigated the characteristics of the electrophysiological brain response, Mismatch Negativity (MMN), in individuals with HD during the premanifest (pHD) and manifest (mHD) stages [99]. The study contrasted the features of the disease with the outcomes of Event-Related Potentials (ERP). MMN recordings were conducted in addition to genetic testing and the evaluation of motor, cognitive, and functional capacities. In the MMN time window, researchers discovered that patients with mHD exhibited a lower Global Field Power (GFP) than those with pHD or Normal Controls (NC). Furthermore, the theta coherence between trials was greater in pHD patients than in mHD patients. These findings indicate that pre-attentive mechanisms are mildly impaired in the pre-symptomatic phase of HD, implying that early functional changes may function as a compensatory measure.

Nunes *et al.* examined the speech characteristics of individuals with HD, prodromal HD, and controls using audio recordings obtained during speech activities [100]. The pausing, intonation, and accuracy of the groups were significantly different. Random forest ML models demonstrated efficient performance in the estimation of medical status, with an Area Under the Curve (AUC) of 0.92 and a balanced accuracy of 73%. The mean absolute errors for the predictions of medical findings ranged from 2.43 to 9.64 percent. These results indicate that voice data can function as a digital meter to facilitate remote illness assessment and monitor HD progression.

Cognitive and behavioral changes are prevalent in the initial phases of HD, as indicated by research conducted by Jellinger *et al.* [101]. In reality, nearly 40% of individuals who have not yet manifested symptoms develop mild cognitive impairments that progress to Alzheimer's disease. Multimodal imaging and transgenic animal models have demonstrated extensive degeneration of white and gray matter, molecular alterations, and synaptic dysfunction. Disruption of prefrontostriatal networks is particularly noteworthy. Synaptopathy is believed to be linked to certain neuropsychiatric and cognitive disorders in significant brain regions, including the hippocampus, striatum, and cerebral cortex. Further research is required to gain a more comprehensive understanding of the early pathophysiological processes to develop biomarkers for early diagnosis and treatment. This research should encompass both neuropathological investigations and neuroimaging.

A mutation in the huntingtin gene results in an unstable expansion of CAG repeats, which in turn causes the huntingtin protein to have an expanded polyglutamine tract, as per Aldous *et al.* [102]. The length of the CAG repeat can be used to predict disease onset. However, the age at which the disease is initiated is also influenced by MSH3 and other genetic modifiers. MSH3 has emerged as a potential therapeutic target owing to its involvement in the expansion of somatic CAG repeats. The removal of Msh3 in a (CAG)185 mutant mouse model resulted in a reduced somatic expansion, but did not affect disease symptoms. This suggests that further expansion may not exacerbate the pathogenesis. This finding underscores the importance of addressing somatic instability during the initial stages of disease progression.

Sampaio *et al.* [103] underscore the significance of high-quality clinical research on HD and the obstacles that have impeded population selection in the past. It introduced the HD Integrated Staging System (HD-ISS), which comprises a biological case definition of HD and a comprehensive staging system for disease progression. The HD-ISS addresses a deficiency in HD research by methodically identifying and classifying patients prior to clinical diagnosis. The presentation of enrichment methodologies within the HD-ISS framework is exemplified by the clinical trial scenarios. In this chapter, the potential for earlier interventional interventions to delay or prevent HD clinical illness is also discussed, and patients and their families may experience the effects of the HD-ISS.

Hassan *et al.* [104] focused on the potential of transportable microwave sensors to address the challenges posed by neurodegenerative diseases, with a particular emphasis on AD. This study describes a new Radiofrequency (RF) antenna that has the potential to detect minute changes in brain dielectric properties associated with atrophy, thereby enabling a non-invasive diagnosis of Alzheimer's disease. The unique RF antenna has the potential to revolutionize the diagnosis and monitoring of neurodegenerative disorders, resulting in substantial improvements in the understanding, identification, and management of these intricate conditions.

Franklin *et al.* [105] commemorated the seminal discovery of the HD gene in 1993, which marked a turning point in genetics and neurology. This discovery was the outcome of decades of collaborative effort, which was motivated by phenomenological discussions and anecdotes from remote South American locations. Throughout the process, over 18,000 blood samples were analyzed by hundreds of academics, resulting in successful mapping and identification of the HTT gene. This discovery has significantly improved our understanding of HD and paved the way for future therapies, in addition to enabling the development of novel molecular tools with significant implications for medical research.

The potential of ML and DL techniques to assist in the diagnosis of HD using Artificial Intelligence (AI) algorithms was the subject of a comprehensive study conducted by Ganesh *et al.* [106]. It highlights current trends, methods, and challenges in the field of HD diagnosis, as well as the use of ML and DL to automate the process using clinical, genetic, and neuroimaging data. This review addresses the potential future research directions, ethical considerations, and limitations in the search for improved HD detection and management. This resource is invaluable for researchers, physicians, and other medical professionals interested in the detection of neurodegenerative diseases using ML and DL.

Zhang *et al.* [107] focused on the development and potential clinical applications of HD biomarkers derived from blood. Biomarker development encompasses a diverse array of processes such as neuronal injury, oxidative stress, endocrine function, immunological responses, metabolism, and differentially expressed microRNAs. Blood-based biomarkers are highly advantageous for early illness detection and monitoring owing to their minimal invasiveness, low cost, and ease of use. Additional research is necessary to standardize the study designs and develop reliable biomarkers for clinical use.

To phenotype mouse models, Bains *et al.* [108] examined the efficacy of automated home-cage surveillance, with a particular emphasis on the climbing activity recorded on wire cage lids. The researchers employed video tracking technology to record a multitude of motor activity data from a variety of inbred mouse strains and the N171-82Q model of HD. Healthy mice exhibited biphasic variations, sexual dimorphism, and age-related decreases on a daily basis. Furthermore, the HD mouse model exhibits signs of motor impairment at an early stage. By integrating the activity at the bottom of the enclosure with climbing behavior, it is possible to characterize mouse strains more accurately. This enables the earlier detection of diseases and the enhancement of repeatability in preclinical investigations.

Stoebner *et al.* [109] investigated cortical abnormalities in HD, including cortical thickness, sulcal depth, and local gyrification index, in addition to the striatum. The local gyrification index indicates differences in insular thickness, whereas cortical thickness indicates variations in the sensorimotor and posterior regions. The fact that these assessments are complementary means they may offer a clinically meaningful indication of disease progression, and abnormalities in the insular regions may indicate early neurodegeneration.

The management of HD is being transformed by AI, as evidenced by the use of predictive analytics and medical image analysis, as per Parekh *et al.* [110]. The utilization of DL and convolutional neural networks enables the identification of biomarkers for early disease detection and for detecting minute changes in the brain. Predictive analytics models are advantageous in the context of treatment decisions and care planning as they assess the progression of illness and anticipate clinical outcomes. Ultimately, the quality of life of HD patients can be enhanced through the implementation of AI in scientific research and healthcare. This could result in more personalized and effective treatment. Close collaboration among healthcare providers, academicians, and technology developers is essential for the successful implementation of AI in HD.

In a study conducted by Kalra *et al.* [111], the occurrence of HD was predicted using computer algorithms and mathematical modelling approaches. This study utilized features from the Global Unified HD Rating Scale (UHDRS) and the TRACK-HD dataset. The predictive models constructed using ML and DL approaches included control patients, pre-manifest gene carriers, early HD patients, and patients in the later stages of HD. These models function as biomarkers that facilitate precise disease monitoring and prognosis. The results indicated that the method was successful in anticipating HD progression.

Alfonso Perez *et al.* [112] describe a biomarker generation approach that uses data on DNA CpG methylation to identify instances of Huntington's Disease. The study effectively distinguished between HD patients and control subjects by reducing the number of CpGs to 237 and utilizing nonlinear methods, such as artificial neural networks. The study employed a limited dataset; however, the results were consistent, suggesting that it could be replicated using a larger dataset. This method demonstrates the value of nonlinear approaches in the evaluation of complex diseases, such as HD, using DNA CpG methylation data.

According to Rosca *et al.* [113], the Montreal Cognitive Assessment (MoCA) can be used to assess cognitive impairment in individuals with HD. 26 studies were identified that encompassed a wide range of comparisons and applications of MoCA in HD. Despite MoCA's strong sensitivity and specificity at the recommended 26-point cutoff, additional cross-sectional research is required to determine the optimal threshold for the diagnosis of HD cognitive impairment. Additional research is necessary to determine the efficacy of MoCA in assessing the cognitive status of patients on HD.

In a mouse model of HD, Podlacha *et al.* [114] discovered a multitude of biomarkers in peripheral blood that are associated with the cognitive and psychological symptoms that are characteristic of the disease. The HD model was developed using a member of the R6/1 rodent family. ELISA assays were performed to quantify hormone and cytokine concentrations in the bloodstream, in addition to mobility and behavioral assessments. Early impairment of anti-inflammatory mechanisms was observed in significantly elevated levels of inflammatory markers (IL-6, TNF-α, IL-1β, and IL-12) during the progression of HD, particularly in females. In addition, there are reports of anxiety and memory issues. This study identified biochemical and behavioral indicators of HD that are easily comprehensible.

The Predict-HD study examined 438 patients with HD gene mutations to gain a more comprehensive understanding of early HD progression before clinical diagnosis (Paulsen *et al.* [115],). We employed nonlinear modelling to establish a correlation between age, CAG repeat length, baseline cognitive, motor, psychological, and imaging factors, and the anticipated time to diagnosis. The majority of indicators underwent substantial changes over one to two decades prior to clinical diagnosis, and the majority were associated with the anticipated time to diagnosis. Our findings offer novel insights into the etiology of HD and suggest prospective biomarkers for HD prevention research.

The PREDICT-HD initiative, led by Jane S. Paulsen, is currently in its ninth year and is dedicated to the examination of individuals with Huntington's disease-causing gene mutations prior to the onset of symptoms [116]. This study elucidated the prodromal stage of the disease and its transition from health to illness. This investigation illuminates the progression of HD and may help establish a framework for comprehending comparable inherited disorders.

Rosas *et al.* [117] have demonstrated the potential of neuroimaging methods in the study of HD by providing objective, noninvasive measures to evaluate the efficacy of therapy and understand the progression of the disease. This review offers a comprehensive summary of the literature on HD neuroimaging and elucidates the potential of these findings as surrogate markers in clinical trials. However, there are also challenges, including the limited availability of subjects and the sensitivity of clinical assessments.

Janice *et al.* [118] conducted a cross-sectional functional Magnetic Resonance Imaging (fMRI). “This study assesses the functional brain alterations in patients with HD gene expansion prior to the onset of HD. Twenty-six preclinical HD (pre-HD) subjects and 13 healthy controls were scanned using functional MRI (fMRI) during a time-replication assignment. The participants were divided into two groups prior to HD based on the estimated onset date: FAR (>12 years to estimated onset) and CLOSE (<12 years to estimated onset). However, the CLOSE group exhibited behavioral abnormalities, striatal atrophy, and reduced neural activation in specific brain areas, whereas the FAR group exhibited altered neural activity patterns, but no substantial atrophy or behavioral changes. The fMRI may be capable of identifying brain dysfunction that occurs up to twelve years before the anticipated onset of HD, according to these findings [118].”

Table **4** provides a comprehensive overview of recent advancements in HD research and therapeutics. Key studies emphasize the importance of early detection and intervention, exploring diverse methodologies ranging from cognitive and behavioral assessments to advanced imaging techniques and genetic analysis. The development of blood-based biomarkers, ML algorithms, and novel therapeutic approaches, such as monoclonal antibodies, showcases multidisciplinary efforts to understand and combat HD. These studies highlight significant strides in identifying presymptomatic markers, enhancing diagnostic accuracy, and improving patient outcomes. Continued research in these areas holds promise for more effective treatment and management strategies for HD patients in the future.

## 8 AMYOTROPHIC LATERAL SCLEROSIS

ALS is a progressive neurodegenerative disease characterized by degeneration of motor neurons, leading to muscle weakness, paralysis, and ultimately, respiratory failure. Despite extensive research, the diagnosis and treatment of ALS remain challenging, necessitating the development of sensitive biomarkers and innovative diagnostic tools. Recent studies have focused on various approaches to enhance early detection and monitor disease progression, including the identification of blood and cerebrospinal fluid biomarkers, advanced imaging techniques, and ML models. This section reviews the latest advancements in ALS research, highlighting the significance of novel biomarkers, the utility of genetic testing, and the potential of ML and imaging in improving diagnostic accuracy and patient outcomes. Figure (**6**) illustrates neuroimaging changes in amyotrophic lateral sclerosis. A: motor cortex (red) atrophy. B: axial T2-FLAIR (fluid attenuated inversion recovery sequence) magnetic resonance image at the level of midbrain, showing hyperintensity in cerebral peduncles corresponding to corticospinal tracts (black arrow).

Turner *et al.* [119] emphasized the importance of sensitive biomarkers for monitoring disease progression and assessing treatment efficacy. Current challenges in the diagnosis and treatment of ALS are the primary focus of this review. Riluzole is the only medication to have demonstrated some efficacy in modifying disease progression across a variety of clinical trials conducted over an extended period. There is potential for a delay between the onset of symptoms and the actual diagnosis if the diagnosis is based solely on clinical assessment. This delay has the potential to result in a missed optimal timeframe for initiation of therapy. The effectiveness of current techniques, including functional rating scales and Forced Vital Capacity (FVC), is limited by specific constraints. By analyzing biomarkers obtained from the blood, cerebrospinal fluid, neuroimaging, and neurophysiological studies, research is currently underway to identify new drug targets, improve diagnostic accuracy, and evaluate treatment efficacy. These biomarkers have the potential to provide valuable insights into the early efficacy of treatments and reduce the need for animal testing by analyzing a variety of observable characteristics in clinical trials.

Grossman *et al.* [120] evaluated a cohort of forty-five ALS patients for changes in disinhibition, executive dysfunction, and apathy using the Frontal Systems Behavior Scale (FrSBe). The findings indicated that apathy is a common behavioral change linked to the onset of illness, regardless of mood. These results indicate a potential correlation between ALS and Frontotemporal Dementia (FTD). Verbal fluency and indifference were significantly correlated. FrSBe was capable of accurately identifying behavioral issues in patients with ALS.

Norel *et al.* [121] suggested speech analysis as a non-intrusive and longitudinal method for assessing the progression of ALS. Speech and motor aptitude tasks, as well as self-reported scores from the ALSFRS-R, were included in the Prize4Life Israel dataset. An ALS mobile analyzer app was employed to gather the data. ML models have been developed to identify the presence and severity of disorders by assessing various acoustic speech factors. 79% of males and 83% of females were accurately classified using leave-five-subjects-out cross-validation, surpassing random chance values.

To improve diagnostic efficiency, Wang *et al.* [122] implemented ML techniques in their investigation to autonomously identify ALS from brief speech samples. The compilation included over 2,500 speech samples collected from 11 patients with ALS and 11 individuals without the disease. The incorporation of articulatory motion information improved the detection performance, as evidenced by the promising results of the leave-subject-out cross-validation.

Zhang *et al.* [123] investigated the feasibility of employing the aggregation of phosphorylated TDP-43 (pTDP-43) as a biomarker for the diagnosis of ALS in muscles that are routinely biopsied. The study included 54 non-ALS controls and 18 ALS patients. In muscle samples obtained from various regions, PTDP-43 aggregation was observed in 94.4% of ALS patients, whereas only 29.6% of control subjects exhibited the same accumulation. The sensitivity and specificity of the semi-quantified pTDP-43 aggregate scoring were 94.4% and 83.3%, respectively, when a cut-off value of 3 was employed. Notably, aggregation of pTDP-43 was observed in patients with ALS prior to the onset of clinical symptoms and electromyographic abnormalities. This finding implies that it has the potential to function as an early diagnostic biomarker for ALS.

TDP-43, a biomarker used to diagnose neurodegenerative diseases, was identified by Turco *et al.* [124]. This study focused on the development of an electrochemical biosensor on a lab-on-a-chip platform. Electro-synthesized poly-pyrrole derivatives with carboxylic groups were employed by the sensor to effectively immobilize TDP-43 antibodies. Differential pulsed voltammetry can be employed to indirectly detect and quantify TDP-43. The chip exhibited high sensitivity, consistent performance, and a Limit of Detection (LOD) of 10 pg/mL within the concentration range of 0.01 ng/mL to 25 ng/mL. In addition, it undergoes a rapid reaction. The potential of this tool as a sensitive point-of-care diagnostic instrument that can facilitate breakthroughs in disease diagnosis and therapeutics is demonstrated by its effective detection in serum samples from both healthy individuals and patients with ALS.

To evaluate the efficacy of gene panel testing with C9orf72 repeat expansion testing in 122 ALS patients, Barel *et al.* [125] conducted a study in Israel. Genetic testing was performed in 85% of 140 patients who were evaluated. Ashkenazi Jewish patients exhibited a C9orf72 frequency of 21%, while non-Ashkenazi medical patients exhibited a frequency of 5.7%. Non-Ashkenazi Jews comprised 14% of the patient population, whereas Ashkenazi Jews, who had disease-causing mutations identified in their gene panels, comprised 5.7%. Panel testing revealed positive results in 12% of the patients with early-onset ALS, even though only 2.9% of the patients with C9orf72 had positive results. The study recommends that C9orf72 be employed as an initial test for Ashkenazi Jewish patients, whereas a gene panel is recommended for non-Ashkenazi individuals and cases of early onset. To enhance our understanding of the disease, it is imperative to conduct a global investigation of the genetic diversity of ALS. The management and treatment of patients using personalized medication will be significantly influenced by this research.

The objective of the study conducted by Simmatis *et al.* [126] was to analyze acoustic speech to identify and categorize ALS patients according to the severity of their illness. To extract 53 acoustic characteristics, a computerized assessment instrument was implemented to analyze speech samples from 22 individuals without health issues and 119 individuals with ALS. The ALS Functional Rating Scale-Revised bulbar score was used to classify the patients into early (ALS-E) and late (ALS-L) phases. Acoustic features could effectively distinguish between ALS-E and ALS-L patients (AUROC = 0.70), ALS patients and controls (AUROC = 0.85), and ALS-E patients and controls (AUROC = 0.78), as indicated by the results of a sparse Bayesian logistic regression classifier. These results suggest that the use of automated sound analysis could be beneficial for the identification and classification of individuals with ALS.

The Cross-Sectional Areas (CSA) of the cervical Vagus Nerve (VN), spinal Accessory Nerve (AN), and Phrenic Nerve (PN) were quantified using high-resolution ultrasonography in a cohort of 80 individuals. The cohort consisted of 40 patients with ALS and 40 healthy controls. Measurements were performed by Walter *et al.* [127]. Prospective and observational methodologies were used for this investigation. Compared with the control group, patients with ALS exhibited a lower Compound Sensory Action Potential (CSA) for all nerves that were examined. Additionally, respiratory function measurements are associated with PN atrophy, enabling the prediction of respiratory impairment. The 1-year survival rate can be predicted by the combination of ALSFRS-R score and Forced Vital Capacity (FVC), which is comparable to the scoring system. The score can also detect neuropathy of cranial nerve motor fibers by incorporating the calibers of the Peripheral Nerve (PN) and Accessory Nerve (AN) motor neurons. Furthermore, the 1-year survival rate in patients with ALS is significantly correlated with the calibers of the PN and AN motor neurons.

To isolate putative biomarkers for ALS, Darabi *et al.* [128] conducted a systematic review. Databases such as PubMed, ScienceDirect, and Web of Science were searched for publications published between January 2015 and June 2023. FUS, miR-27a-3p, SOD1, TDP-43, pTDP-43, and CORO1A were identified as biomarkers in six studies that examined fluid-based exosomal biomarkers. Plasma exosomes have shown predictive biomarkers, including NFL and TDP-43, despite the suggestion that downregulation of miR-27a-3p serves as a diagnostic indicator. It has been suggested that the expression of CORO1A is elevated in order to track disease progression. A panel approach is essential because it has been determined that no single biomarker is sufficient. Exosome detection instruments, including Exo-Flow, Exo-Easy, ExoQuick, Exo-spin, ME kit, and ExoQuick Plus, are capable of detecting cerebrospinal fluid and blood.

Donohue *et al.* [129] conducted a study using coupled data from the Speech Intelligibility Test (SIT) and the ALS Functional Rating Scale-Revised (ALSFRS-R). The sensitivity and specificity of the ALSFRS-R bulbar subscale and speech items in diagnosing dysarthria in patients with ALS were evaluated to evaluate their accuracy. Dysarthria was confirmed in 72.4% of cases, and those with dysarthria achieved reduced scores on the speech and bulbar subscales of the ALSFRS-R. The bulbar and speech items exhibited an AUC of 0.81, specificities of 57% and 75%, and sensitivities of 86% and 79%, respectively. In general, these findings suggest that the speech and bulbar subscales of the ALSFRS-R have a moderate capacity to identify dysarthria in ALS patients.

Wallace [130] developed a biosensor that detects full-length phosphorylated TDP-43 (pTDP-43), a biomarker associated with severe acute lung damage, at an early stage. The biosensor was based on Electrochemical Impedance Spectroscopy (EIS). Protein recognition assays were employed by the biosensor to identify proteins, specifically TDP-43 Antibodies (Abs), immobilized on Gold (Au) surfaces. Surface characterization of the Ab-Au system using EIS demonstrated that the charge-transfer resistance (Rct) exhibited significant fluctuations in response to fluctuations in protein concentration, both prior to and following pTDP-43 binding. The detection of pTDP-43 was highly effective in distinguishing it from common proteins, such as bovine serum albumin, owing to its selectivity. Nevertheless, the detection sensitivity depended on the specific antibody employed, with a detection limit of 11 ± 6 nM. This method provides a feasible approach for identifying biomarkers associated with ALS, as it can be modified to identify a variety of disease-related biomarkers.

Wu *et al.* [131] conducted a study in Taiwan to analyze clinical data from a population-based database in order to gain a more comprehensive understanding of the temporal dynamics of ALS progression and its interaction with related illnesses. To achieve this, they employed a primary tree-based model. Eight distinct trajectories were observed in patients with ALS, each of which represented a distinctive pattern of illness linkage during various phases of the disease. This research provided innovative insights into the disease mechanisms and risk factors associated with ALS by reimagining disease progression as a sequential progression through multiple phases. These discoveries can enhance the overall well-being and prognosis of patients by enabling the development of personalized treatment strategies.

Hippocampal Magnetic Resonance Spectroscopy (MRS) was comprehensively evaluated in patients with non-demented ALS who performed well on memory tests by Christidi *et al.* [132]. The objective of this study was to investigate the metabolic indicators in the frontotemporal region and their relationship to cognitive function. In addition to clinical and cognitive assessments, patients were subjected to a diverse array of imaging techniques such as MRS, high-resolution structural imaging, and Diffusion Tensor Imaging (DTI). There were no discernible variations in the volumetric or diffusivity measurements. Nevertheless, the hippocampi of individuals with ALS exhibited elevated levels of metabolites, including tNAA, tNAA/tCr, and tCho, in both hemispheres of the brain. Improved memory function is associated with higher levels of tNAA/tCr, whereas prolonged symptom duration and increased impairment are associated with higher levels of tCho. These results indicate that Hippocampal MRS can be a valuable biomarker for evaluating the extra-motor disease burden in ALS. In addition, this information can be used to guide future clinical investigations and applications.

Udine *et al.* [133] conducted an exhaustive review that employed various methods to identify genes associated with ALS. The discovery of novel genes has been significantly influenced by modern approaches, such as genome-wide association studies and Next-Generation Sequencing (NGS), and traditional techniques, such as Sanger sequencing and linkage analysis. Long-read sequencing has the potential to uncover structural variations and repetitive expansions, thereby addressing the intricate genetics of ALS. This research not only underscores the significance of recently developed long-read sequencing technologies in the identification of genes but also examines the ways in which these methods enhance our understanding of the genetics of ALS.

Vidovic, Maximilian, *et al.* [134] ALS is a condition that necessitates early detection. This investigation also encompasses an assessment of existing diagnostic procedures and potential future developments. In the past, diagnosis was established through clinical observations, laboratory data, and electrophysiological testing. The utilization of fluid biomarkers, such as neurofilaments, and advancements in imaging techniques have enabled early detection of diseases and enhancement of precision. Furthermore, genetic testing is now more easily accessible, allowing for the identification of genetic abnormalities that are detrimental to health before symptoms appear. Currently, patients have access to comprehensive prognostic information through customized survival prediction models. The primary goal of this evaluation was to improve the ALS diagnosis process by implementing timely interventions and attaining better patient outcomes.

In a retrospective case-control study, Truffert *et al.* [135] compared the motor evoked potentials (T-MEPs) of 97 ALS patients with those of 60 matched controls, as well as tendon reflex recordings. Central motor conduction time demonstrated high sensitivity (82%) and specificity (93%) for the identification of upper motor neuron dysfunction, surpassing the diagnostic accuracy of clinical examination. The T response to the MEP response amplitude ratio (T/MEP ar) is more sensitive for diagnosing ALS and for detecting aberrant hyperreflexia. T-MEPs can accurately identify asymmetries overlooked during clinical examinations. This study demonstrates the effectiveness of T-MEPs in detecting abnormalities in corticospinal conduction in ALS and facilitating the diagnostic process.

This publication presents a study conducted by Gomes *et al.* [136] that focuses on the identification of early indicators of ALS by analyzing facial expressions using Facial Point Graphs. Comparisons of facial muscle movements during specific tasks, such as extending the mouth, can be employed to distinguish between ALS patients and healthy individuals. This strategy outperformed the other methods when evaluated using the Toronto Neuroface dataset. These discoveries support the use of computer analysis of facial images as a viable method for the early detection of ALS.

Shabber *et al.* [137] conducted a study that examined the use of ML techniques in the diagnosis of ALS by analyzing speech impairment recordings. This study aimed to classify individuals with ALS and Healthy Controls (HC) by utilizing datasets that contained speech recordings from patients with ALS and healthy individuals. An SVM classifier obtained a classification accuracy of 98.5% by utilizing characteristics such as shimmers and jitter extracted from extended vowel phonation. These results underscore the potential of ML to facilitate early identification of ALS and to inform future research objectives in this field.

In addition, Choayb *et al.* [138] conducted a case study on a 64-year-old woman who had been diagnosed with ALS. The patient exhibited typical MRI findings, such as brilliant tongue and motor band signs, which suggest that the upper motor neurons are dysfunctional. Furthermore, the MRI results provide essential diagnostic information that complements electromyography's capacity to identify lower motor neuron involvement in ALS. This case illustrates the significance of MRI for the precise diagnosis and monitoring of ALS progression.

In addition, Nishikawa *et al.* [139] compared Motor Unit (MU) abnormalities between individuals with ALS and a control group in their study. HD-SEMG was implemented in this investigation. The investigation involved 16 control volunteers and 16 ALS patients who performed contractions at 30% of their maximal voluntary contraction. High-Density Surface Electromyography (HD-SEMG) signals of the vastus lateralis muscle were analyzed. The findings suggest that patients with ALS exhibited substantially higher values (p < 0.001) for the following parameters: mean firing rate, recruitment threshold, MU firing rate at recruitment, coefficient of variation, and motor neuron excitability. Disease severity significantly correlated with several MU features (p < 0.001). Multivariable analysis demonstrated a substantial independent correlation between elevated MU discharge rate during recruitment and ALS. The results of our investigation suggest that neurodegeneration induces compensatory Motor Unit (MU) activity. The study also found that individuals with ALS exhibited increased excitability during the recruitment process.

In addition, Tena *et al.* [140] conducted a study on the deterioration of speech to establish a sophisticated automated method for diagnosing bulbar involvement in patients with ALS. A set of 50 phonatory-subsystem and time-frequency parameters was extracted from a dataset that included recordings of five Spanish vowels emitted by both male and female speakers. Multiple supervised classification models were assessed, and significant features were identified using a Multivariate Analysis of Variance. In comparison to previous models, the random forest approach employed in this study resulted in substantially higher accuracy rates (98.01% for females and 96.10% for males). The prediction capabilities were considerably improved by the inclusion of time-frequency variables, particularly when the genders were analyzed separately. The proposed method has the potential to be integrated into various recording devices, including smartphones.

Fournier *et al.* [141] emphasized the significance of meticulous clinical trial design in the identification of ALS treatments. The significance of validating biomarkers, employing statistical enrichment methods, and utilizing appropriate outcome measurements has been underscored in the text. It is imperative to involve patients with ALS as advisors and advocates to enhance the design of the study and retention of participants. The report also discusses the obstacles associated with the expansion of the availability of experimental treatments and promotion of patient autonomy. In conclusion, this study underscores the significance of employing comprehensive trial design methodologies that achieve a harmonious balance between the prioritization of patient interests, applicability to a broader population, and statistical efficacy. No specific dataset or methodology was presented in the summary.

Mastrogiovanni *et al.* [142] examined the plasma oxylipin profiles of 74 patients with ALS and controls to determine the significance of the oxylipin profile in the regulation of inflammation. Oxylipins produced from linoleic acid, specifically 9-HODE and 13-HODE, exhibited a substantial decrease in patients with ALS. The concentration of 5-hydroxy-eicosatetraenoic acid and other 5-lipoxygenase products was also reduced. Isoprostanes from the F2α family are exclusively present in the blood of ALS patients. Nevertheless, no specific mediators that promoted resolution were identified. Individuals with ALS demonstrated a decrease in the quantity of 14-hydroxy-docosahexaenoic acid, which is a marker for resin synthesis. This suggests a malfunction in the resolution of inflammation. The duration of illness was positively correlated with the concentrations of 9- and 13-HODE in blood plasma. The investigation of oxylipins in human plasma conducted in this study offers a glimpse of the pathogenesis of ALS.

To identify fasciculations, Fukushima *et al.* [143] employed Muscle Ultrasonography (MUS) to assess 100 ALS patients, 50 of whom were in the early phases of the disease, and 100 individuals without ALS. Hierarchical clustering and machine learning techniques are required for the development of diagnostic models. The models utilized in this study, which involved eight muscles in four body locations, exhibited significant sensitivity, specificity, and positive predictive value in patients with both early- and late-stage ALS. ALS diagnosis was found to have low sensitivity but high specificity when fasciculations were observed in the thoracic or cranial regions. In general, diagnostic models based on MUS-fasciculation have demonstrated promising accuracy in the identification of the early stages of ALS.

As shown in Table **5**, significant progress has been made in ALS research, particularly in diagnostic techniques, biomarkers, and ML approaches. Recent studies have underscored the utility of diverse methodologies, from advanced imaging and biomarker identification to innovative ML models, to enhance diagnostic accuracy and monitor disease progression. For example, the integration of speech analysis and high-resolution ultrasonography offers new insights into early disease detection and progression. The development of novel biomarkers and sensitive biosensors will further contribute to improving diagnostic precision and treatment efficacy. Collectively, these advancements reflect a multifaceted approach towards understanding and managing ALS, highlighting both the challenges and promising solutions in the ongoing quest for effective treatment and early diagnosis.

## 9 CHALLENGES AND LIMITATIONS IN CURRENT COMPUTATIONAL APPROACHES

The integration of computational approaches into brain disease diagnosis has brought remarkable advancements; however, several challenges and limitations continue to impact their effectiveness and application. In this section, we discuss these issues in detail.

### 9.1 Data Quality and Heterogeneity

One of the primary challenges is the variability and quality of the data used in computational models. Brain disease datasets are often obtained from diverse sources, each with its own set of imaging protocols, patient demographics, and diagnostic criteria. This heterogeneity can lead to inconsistencies in the data, affecting the performance and generalizability of computational models. For instance, differences in MRI scanners, imaging protocols, or even patient positioning can introduce variability that complicates data integration and model training.

### 9.2 Interpretability and Transparency

Many advanced computational methods, particularly DL models, function as “black boxes.” Although these models can achieve high accuracy, their decision-making processes are not easily interpretable. This lack of transparency poses a significant challenge in clinical settings where understanding how a model arrives at its conclusions is crucial for trust and adoption. Clinicians must interpret model predictions within the context of patient-specific information, making it essential to develop models that provide insights into their reasoning.

### 9.3 Generalization and Overfitting

Computational models trained on specific datasets may struggle to generalize to new, unseen data, particularly if the training data are not representative of the broader population. Overfitting occurs when a model learns to perform well on training data, but fails to maintain accuracy on independent test datasets. This issue is particularly relevant in brain disease diagnosis, in which patient variability and disease progression can significantly affect the model performance.

### 9.4 Computational and Resource Demands

Advanced computational techniques such as DL often require substantial computational resources, including high-performance GPUs and large memory capacities. The resource-intensive nature of these methods limits their accessibility and feasibility, particularly in resource-constrained settings. Additionally, the time required for training and fine-tuning models can be prohibitive, thus delaying their deployment in clinical practice.

### 9.5 Data Privacy and Ethical Considerations

The use of sensitive patient data for training and validating computational models raises privacy and ethics concerns. Ensuring that the data are anonymized and used responsibly is crucial for maintaining patient trust and complying with legal and ethical standards. Researchers and developers must navigate complex regulations and ethical considerations to ensure that patient data are handled appropriately and that models do not inadvertently introduce biases.

### 9.6 Integration into Clinical Practice

The integration of computational approaches into the existing clinical workflows presents several challenges. Issues such as interoperability with Electronic Health Records (EHRs), clinician training, and workflow compatibility must be addressed to ensure that these tools are effectively utilized in practice. Successful integration requires collaboration among computational scientists, clinicians, and IT professionals to develop solutions that fit seamlessly into clinical environments.

### 9.7 Validation and Standardization

The lack of standardized protocols for model validation and performance assessment complicates the comparison of different computational approaches. Without agreed-upon benchmarks and validation metrics, it is challenging to evaluate the relative merits of various models and to ensure their reliability across different settings. Establishing standardized evaluation criteria and protocols is essential for advancing the field and facilitating the adoption of computational tools.

### 9.8 Cost and Accessibility

The cost of developing and implementing advanced computational tools is therefore significant. This includes the expenses related to data acquisition, computational resources, and software development. High costs can limit the accessibility of these technologies, particularly in low-resource or underserved regions. Ensuring that computational approaches are affordable and widely accessible is essential for broader adoption and impact.

Addressing these challenges requires ongoing research, interdisciplinary collaboration, and the development of innovative solutions. By overcoming these limitations, computational approaches can realize their full potential for improving the diagnosis and management of brain diseases, ultimately enhancing patient care and outcomes.

## 10 EXPLORATION OF POTENTIAL ADVANCEMENTS IN COMPUTATIONAL APPROACHES FOR BRAIN DISEASE DIAGNOSIS

The field of computational approaches to brain disease diagnosis is rapidly evolving, driven by advancements in technology and an increased understanding of brain disorders. This section explores potential advancements and discusses emerging technologies poised to revolutionize the diagnostic landscape.

### 10.1 DL and Artificial Intelligence

DL, a subset of AI, has shown considerable promise for enhancing brain disease diagnosis. Techniques such as CNNs and RNNs are increasingly being used to analyze neuroimaging data, detect subtle patterns, and predict disease progression. These models handle high-dimensional data and can uncover intricate relationships that traditional methods may miss. Future advancements in DL could lead to more accurate and early diagnoses, as well as personalized treatment plans tailored to individual patient needs.

### 10.2 Multi-Modal Data Integration

Integrating multimodal data—such as neuroimaging, genetic, electrophysiological, and clinical information—offers great potential for enhancing diagnostic accuracy. Advanced algorithms are being developed to synthesize and analyze data from different modalities, thereby providing a more comprehensive view of brain diseases. For example, combining MRI scans with Electroencephalography (EEG) data can enhance the detection of epileptic seizures or identify biomarkers for neurodegenerative diseases. Future research should focus on refining these integration techniques to offer a holistic diagnostic approach.

### 10.3 Precision Medicine and Genomics

The advent of genomics and precision medicine has transformed this approach to the diagnosis of brain diseases. By analyzing genetic data, researchers can identify genetic markers and variations associated with specific brain disorders. Computational models that incorporate genomic data can predict disease risk, personalize treatment strategies, and identify potential therapeutic targets. Advances in genome sequencing technologies and bioinformatics will further enhance the ability to link genetic information with clinical outcomes, leading to more precise and individualized diagnostic and therapeutic approaches.

### 10.4 Brain-Computer Interfaces (BCIs)

Brain-Computer Interfaces (BCIs) represent cutting-edge technology with the potential to revolutionize the diagnosis and treatment of brain diseases. BCIs facilitate direct communication between the brain and external devices, enabling the real-time monitoring of neural activity. In the context of brain diseases, BCIs can be used to track disease progression, assess treatment efficacy, and provide adaptive interventions based on a patient's neural signals. Ongoing research aims to improve the accuracy, usability, and clinical applicability of BCIs for the diagnosis and management of brain disorders.

### 10.5 Advanced Neuroimaging Techniques

Emerging neuroimaging technologies, such as functional MRI (fMRI), with enhanced spatial and temporal resolution, provide new insights into brain function and pathology. Techniques, such as Diffusion Tensor Imaging (DTI) and Magnetoencephalography (MEG), offer detailed information about brain connectivity and activity. Future advancements in neuroimaging could lead to earlier and more precise detection of brain diseases as well as a better understanding of their underlying mechanisms.

### 10.6 Big Data and Cloud Computing

The rise of big data and cloud computing is transforming the field of brain-disease diagnosis. Large-scale datasets can be stored, processed, and analyzed using cloud-based platforms, enabling researchers to access and share data more efficiently. Big data analytics allows the identification of patterns and trends across vast datasets, leading to new insights and potential diagnostic biomarkers. Cloud computing also facilitates collaboration between researchers and clinicians, accelerating the development and dissemination of new diagnostic tools.

### 10.7 Personalized and Adaptive Algorithms

Personalized and adaptive algorithms are emerging as promising approaches to improve diagnostic accuracy and patient outcomes. These algorithms can adjust their predictions and recommendations based on the individual patient data and responses. For instance, adaptive models can tailor diagnostic criteria and treatment plans to the specific characteristics of each patient, enhancing the precision and effectiveness of interventions.

### 10.8 Ethical and Regulatory Considerations

As computational approaches have advanced, ethical and regulatory considerations have become increasingly important. Ensuring that new technologies are developed and implemented in a manner that respects patient privacy, avoids bias, and adheres to regulatory standards is crucial. Future advancements are needed to address these concerns to ensure that emerging technologies are both effective and ethical.

In summary, the exploration of potential advancements in computational approaches to brain disease diagnosis highlights a range of exciting developments. These emerging technologies promise to enhance diagnostic accuracy, personalize treatment, and improve patient outcomes. Ongoing research and innovation will continue to drive progress in this field, offering new opportunities to address the challenges posed by brain disease.

## 11 SUMMARY OF KEY FINDINGS AND FUTURE DIRECTIONS

- Summary of Key Findings

A review of computational approaches to brain disease diagnosis revealed several critical insights into the current state of the field.

- **Diverse Methodologies:** This field utilizes a broad spectrum of computational techniques, including ML, DL, neuroimaging analysis, and electrophysiological signal processing. Each method offers unique advantages, such as pattern recognition capabilities and high-dimensional data handling, which contribute to the early and accurate diagnosis of brain diseases.
- **Enhanced Diagnostic Precision:** Advances in computational methods have significantly enhanced diagnostic accuracy. ML algorithms, particularly those utilizing DL models, have demonstrated strong performance in identifying subtle patterns in neuroimaging data and in predicting disease progression. This has led to improved early detection and more precise classification of brain disorders.
- **Integration of multimodal data:** The integration of data from various sources, such as neuroimaging, genetic information, and electrophysiological signals, has emerged as a powerful approach for improving diagnostic precision. Multimodal integration allows for a more comprehensive assessment of brain diseases, facilitating earlier detection and more accurate differentiation between conditions.
- **Emerging Technologies:** This field is witnessing the rise of several emerging technologies, including Brain-Computer Interfaces (BCIs), advanced neuroimaging techniques, and precision medicine approaches. These technologies have the potential to revolutionize brain disease diagnosis by providing real-time monitoring, detailed insights into brain functions, and personalized treatment strategies.
- **Challenges and Limitations:** Despite significant progress, several challenges have persisted. Issues such as the need for large and diverse datasets, model interpretability, and computational resource demands continue to affect the effectiveness and clinical implementation of computational approaches. Addressing these challenges is crucial for advancing the field.
  - Future Directions.
- **Advancements in DL Models:** Future research should focus on refining DL models to enhance their interpretability and reduce their computational demands. Innovations in model architectures and training algorithms could improve the diagnostic accuracy and make these models more accessible for clinical use.
- **Enhanced Multimodal Data Integration:** Continued development in multimodal data integration techniques is needed to combine neuroimaging, genetic, and electrophysiological data more effectively. Research should aim to create robust algorithms that are capable of synthesizing diverse data sources to provide a comprehensive diagnostic overview.
- **Expansion of Precision Medicine:** The Integration of genomics and personalized medicine into computational diagnostics holds great promise. Future work should focus on leveraging genetic data to tailor diagnostic and therapeutic approaches to individual patients and enhance the precision and effectiveness of interventions.
- **Advancements in Neuroimaging Techniques**: Ongoing innovations in neuroimaging technologies will be crucial for detecting brain diseases at earlier stages. Future advancements should aim to improve the imaging resolution and develop new techniques that offer deeper insights into brain function and pathology.
- **Development of Brain–Computer Interfaces**: Research into BCIs should continue to advance, with a focus on improving their accuracy, usability, and clinical applicability. BCIs have the potential to transform disease monitoring and intervention, making the real-time tracking of brain activity a practical reality.
- **Big Data and Cloud Computing:** Leveraging big data analytics and cloud computing can enhance the ability to handle and analyze large datasets. Future efforts should focus on developing scalable solutions that facilitate data sharing, collaboration, and the development of new diagnostic tools.
- **Ethical and Regulatory Considerations**: As new technologies emerge, it is essential to address ethical and regulatory issues to ensure responsible implementation. Research should focus on developing guidelines and frameworks that protect patient privacy, ensure fairness, and comply with regulatory standards.

## CONCLUSION

This review highlights the progress made in computational methods for brain disease diagnosis. While significant advancements have been achieved, addressing ongoing challenges such as data limitations and method validation is essential. Future research should focus on overcoming these hurdles to develop more accurate, early, and personalized diagnostic and therapeutic strategies.

## Figures and Tables

**Fig. (1):**
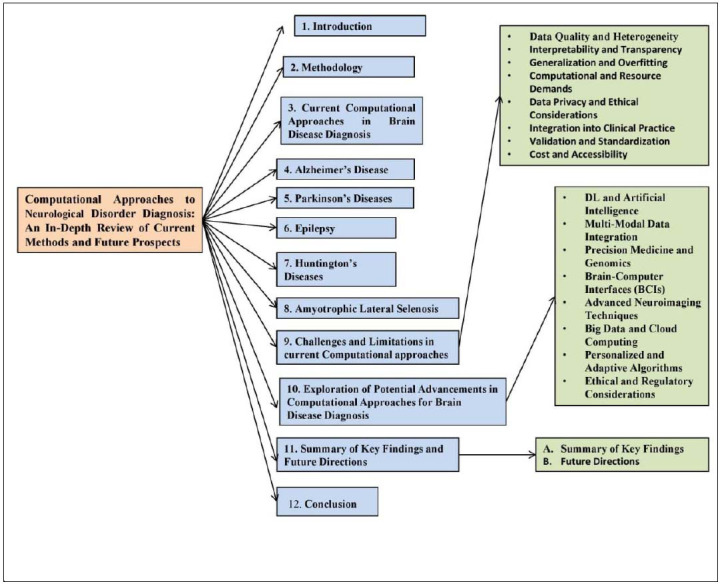
Overview structural diagram of the review framework.

**Fig. (2):**
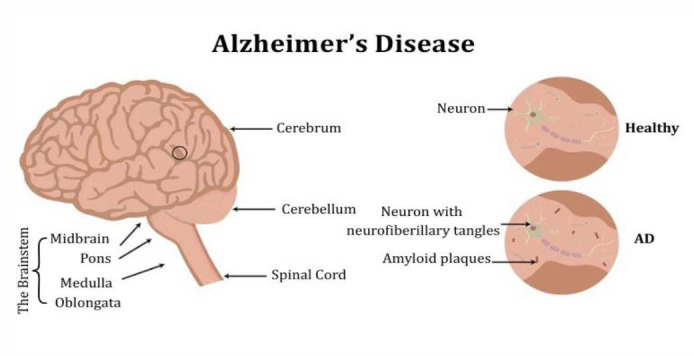
Illustration of healthy brain tissue and tissue affected by alzheimer's.

**Fig. (3):**
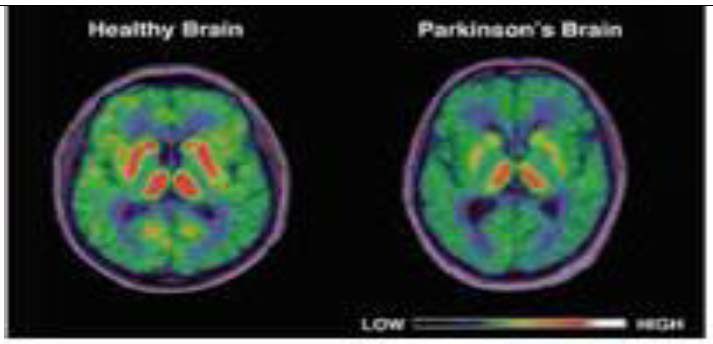
PET scan of a healthy and Parkinson’s brain.

**Fig. (4):**
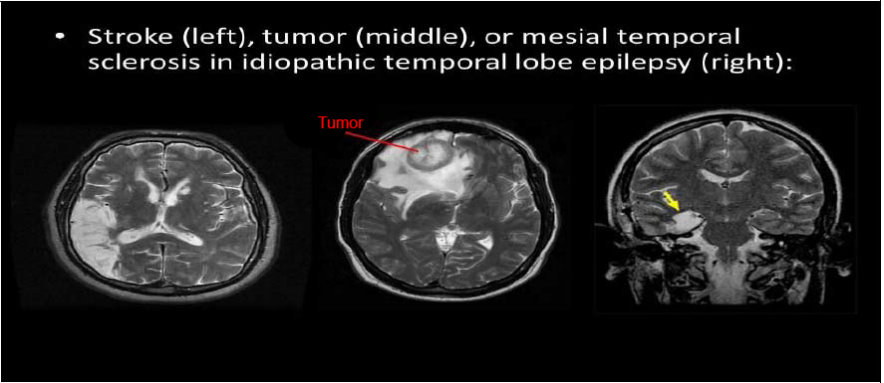
Abnormal brain MRI in epilepsy.

**Fig. (5):**
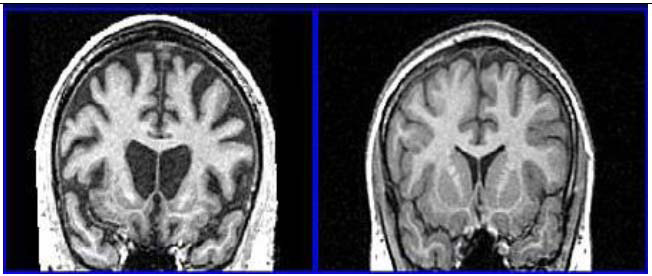
MRI scans of a huntington’s disease (HD) sufferer’s brain (left) and a normal brain (right).

**Fig. (6):**
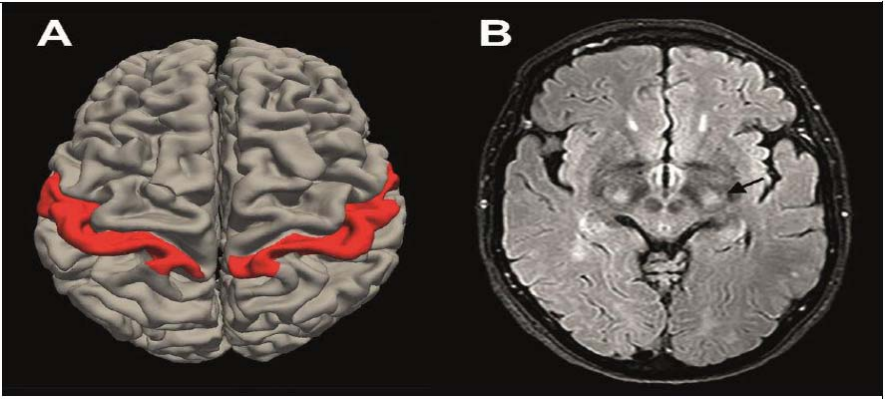
Neuroimaging changes in amyotrophic lateral sclerosis. **A**: motor cortex (red) atrophy. **B**: axial T2-FLAIR (fluid attenuated inversion recovery sequence) magnetic resonance image at the level of midbrain, showing hyperintensity in cerebral peduncles corresponding to corticospinal tracts (black arrow). “Adapted from Ilieva, H. *et al.* [144]”.

**Table 1:** Overview of state-of-the-art techniques for early detection and classification of Alzheimer's disease.

**Study/Refs.**	**Methodology**	**Dataset Used**	**Key Findings and Advantages**
Marwa EL-Geneedy, *et al.* [8]	DL, CNN, MRI	Alzheimer’s classification dataset	Achieved 99.68% accuracy in classifying AD stages, highlighting the effectiveness of CNN architectures in processing MRI data.
Chimthanawala, *et al.* [9]	Noninvasive Biomarkers	Not specified	Highlights strong correlation between brain pathology and biomarkers for early AD detection, emphasizing the need for noninvasive methods.
Therriault, *et al.* [10]	Immunoassay, Mass Spectrometry, CSF	Cerebrospinal Fluid biosamples	Immunoassays showed slightly better performance than mass spectrometry for detecting phosphorylated tau biomarkers, suggesting a reliable diagnostic approach.
Prasath, *et al.* [11]	Pipelined LeNet (PLN) Architecture, MRI	MRI datasets	High classification accuracy with PLN design improves efficiency and diagnostic speed, particularly beneficial for low-resolution imaging.
Illakiya, *et al.* [12]	DL, Neuroimaging, CNN, RNN, TL	Mixed neuroimaging datasets	Emphasized the impact of DL on AD detection and identified gaps in biomarker research, signaling the need for comprehensive studies on predictive models.
Vrahatis, Aristidis G., *et al.* [13]	Non-invasive Biomarkers, AI	Not specified	Emphasized the roles of AI and DL in processing large, noninvasive datasets, suggesting potential for early AD detection.
Aberathne, *et al.* [14]	Longitudinal Data Analysis, ML, MRI, PET	Longitudinal neuroimaging datasets	Showed that ML combined with MRI and PET can predict AD progression, supporting continued exploration of temporal changes in neuroanatomy.
Marwa EL-Geneedy, *et al.* [8]	DL, MRI	Alzheimer’s classification dataset	Proposed a unique DL approach leveraging MRI data, outperforming previous diagnostic methods with enhanced accuracy.
Petti, Ulla, *et al.* [15]	Ethical Implications, AI, Speech	Not applicable	Highlights the need for ethical frameworks in AI systems for AD detection, stressing patient confidentiality and informed consent.
Lu, Dongwan, *et al.* [16]	Salp Swarm Algorithm, Fuzzy K-nearest Neighbors	Not specified	Improved classification performance through innovative optimization techniques, demonstrating advancements in traditional ML methods.
Ganesh, D., *et al.* [17]	CNN, Neuroimaging	MRI and PET datasets	Showed CNNs effectively distinguish AD patients from healthy individuals, marking a significant advancement in early detection methods.
Wang, Ping, *et al.* [18]	Electrochemical Sensing, Gold Nanostars, EIS	Amyloid-beta samples	Developed a highly sensitive detection method for amyloid-beta oligomers, facilitating potential point-of-care diagnostics.
Helaly, *et al.* [19]	CNN, Transfer Learning, Web Application	Medical image datasets	Achieved high accuracy in classifying AD stages, demonstrating effectiveness in both 2D and 3D imaging techniques.
Arafa *et al.* [20]	DL, MRI, PET	Mixed neuroimaging datasets	Reviewed advancements in DL for AD detection, showcasing improvements in classification accuracy for early intervention.
Rafii, *et al.* [21]	Plasma Biomarker Assays, Recruitment Strategies	Clinical trial datasets	Discussed advancements in preclinical trials and the potential for anti-amyloid therapies as preventative measures against cognitive decline.
Cheung, Carol Y., *et al.* [22]	DL, Retinal Images	Retinal imaging datasets	Confirmed retinal images as a promising noninvasive screening tool for AD detection and dementia with high accuracy.
Kong, Zhaokai, *et al.* [23]	MRI, PET, 3D Convolutional Neural Networks	AD Neuroimaging Initiative (ADNI) dataset	Improved classification tasks with fused images, outperforming previous methods in specificity and accuracy.
Gao, Shuangshuang, *et al.* [24]	DL, Biomarkers, Feature Extraction	Mixed datasets	Emphasized DL’s superior efficacy in AD detection, combining various methodologies and models for comprehensive analysis.
Helaly, *et al.* [25]	DL-AHS, U-Net, ResNet, MRI	ADNI and NITRIC datasets	Proposed an advanced framework for hippocampus segmentation, achieving high accuracy for AD diagnosis.
Orouskhani, Maysam, *et al.* [26]	Few-Shot Learning, Deep Triplet Network, VGG16	OASIS dataset	Addressed the limited sample size issue in AD diagnosis with improved accuracy using a novel deep triplet network architecture.
Venugopalan, Janani, *et al.* [27]	DL, MRI, SNPs, Clinical Tests	ADNI dataset	Utilized multimodal data for enhanced classification of AD stages, significantly improving performance over traditional models like SVM.
Abhilash *et al.* [28]	Computer-Assisted Diagnostics, SVM	Mixed imaging datasets	Achieved high precision in early AD detection using various imaging analyses, demonstrating the potential of computer-assisted methods.
Ebrahimi *et al.* [29]	AI, Sequence-Based Models, ResNet-18, TCN	3D MRI volumes	Improved classification accuracy by integrating CNNs with sequence-based models, with the TCN showing the best performance metrics.
Al-Shoukry, *et al.* [30]	DL	Not specified	Highlighted the potential for DL in early AD detection while noting the current absence of standardized methodologies.
Safi, *et al.* [31]	EEG Data, Signal Decomposition, KNN, DWT	EEG datasets	Achieved 97.64% accuracy in early AD detection through the application of advanced signal processing techniques combined with classification algorithms.
Liu *et al.* [32]	Deep Separable CNN, Mobile Embedded Systems	OASIS MRI dataset	Developed a lightweight model appropriate for mobile systems, effectively identifying AD while minimizing resource consumption.

**Table 2:** Summary of state-of-the-art methods for PD diagnosis and management.

**Authors and Refs.**	Methodology	Dataset	Computational Techniques	Key Results
Ali, Liaqat *et al.* [33]	Regularized Linear SVM and Deep Neural Network	Voice recording-based datasets	SVM for feature refinement, DNN for classification	Achieved 100% accuracy (leave-one-out CV), 97.5% accuracy (k-fold CV), showcasing non-invasive diagnostic potential
Islam, Md Ariful *et al.* [6]	ML and DL techniques	Speech, handwriting, and wave spiral datasets	Various ML/DL classifiers	Enhanced diagnostic precision; offers a comprehensive insight for clinical decision-making
Cuk, Aleksa *et al.* [34]	LSTM with attention and enhanced COA	Real-world clinical gait dataset	LSTM for sequential data processing; COA for optimization	Achieved 87.4187% accuracy in gait analysis for PD detection
Veetil, Iswarya Kannoth *et al.* [35]	Variational Mode Decomposition on sustained phonations	Spanish and Italian speech datasets	DL for feature extraction and classification	Cross-lingual accuracy: 65%-80%, within dataset: 90%-95%, independent dataset: 63%, demonstrating robustness across languages
Chen, Qian *et al.* [36]	Magnesium-based micromotors for α-syn detection	Bloodstream samples	Micromotor technology with electrochemical detection	Significant enhancement in detecting α-syn, crucial for early PD detection
Sun *et al.* [37]	Fluorescence imaging techniques	Various biomarkers	Targeted fluorescent probes for biomarker study	Contributed valuable insights into PD pathology and probe development
Cantürk *et al.* [38]	AI using scalogram images and DL	Voice signals	Scalogram images processed *via* deep learning	Achieved 95% accuracy using deep feature fusion techniques for voice analysis
Rajinikanth *et al.* [39]	Pretrained Lightweight Deep-Learning (PLDL)	Hand sketching data	KNN for classification, DL for feature extraction	Achieved 100% accuracy, demonstrating high efficacy in diagnosing PD from sketches
Bakkialakshmi, V. S. *et al.* [40]	Deep learning on speech signal features	Dataset with speech signal features from 3000 individuals	Various DL models (ResNet50, VGG16, *etc.*)	Encouraging accuracies (89%-95%), with AlexNet achieving the highest accuracy of 95%
Höglinger, Günter U. *et al.* [41]	SynNeurGe based on biological factors	Clinical and biological datasets	Integrating genetic and clinical data for diagnosis	Aims for precision medicine and disease-modifying treatments in PD
Govindu *et al.* [42]	ML algorithms in telemedicine	MDVP audio data from 30 individuals	SVM, RF, KNN, and LR for audio analysis	Random Forest achieved 91.83% accuracy and 0.95 sensitivity, showcasing telemedicine's potential in PD detection
Gupta, Rohan *et al.* [3]	AI and ML on various characteristics	Neuroimaging, speech, and gait data	ML for pattern recognition and management	Highlighted the significant potential of integrated AI and ML for early PD detection and management
Sayed, Md Abu *et al.* [43]	Advanced ML techniques on voice features	Various voice datasets	XGBoost, LightGBM, SVM, and AdaBoost	LightGBM achieved 96% accuracy and 100% sensitivity, underscoring voice analysis for timely PD detection
Concha-Marambio, Luis *et al.* [44]	αSyn-Seed Amplification Assay (αS-SAA)	Cerebrospinal fluid samples	Assay for detecting α-synuclein	Achieved 98% accuracy in PD-CSF detection, highlighting its utility in early diagnosis
Alalayah, Khaled M. *et al.* [45]	RFE, t-SNE, PCA with classifiers	Auditory signals	RFE for feature selection; t-SNE/PCA for dimensionality reduction	RF with t-SNE achieved 97%, MLP with PCA achieved 98% accuracy, improving prior results
Wu, Peng *et al.* [46]	AI-based gait assessment	Gait data	AI algorithms for real-time evaluation	Improved motor independence and diagnostic precision for PD patients through ongoing gait monitoring
Wang, Rui *et al.* [47]	Various detection methods for α-Syn	Multiple sources, including clinical data	Mass spectrometry, antigen-antibody detection, and electrochemical sensors	Early detection of α-syn aggregates aids in monitoring disease progression and intervention strategies
Mahmood, Awais *et al.* [48]	Extraction of vocal characteristics	Voice samples	Technique for analyzing vocal characteristics for PD diagnosis	Achieved RMSE error of 0.10; provides a standardized method for monitoring PD progression
Parajuli *et al.* [49]	Deep Learning on EEG time series	Sleep EEG data from PD patients	CNN and Variational Mode Decomposition (VMD)	VMD-based model achieved >99% accuracy, sensitivity, and specificity in detecting Mild Cognitive Impairment (MCI) in PD
Abdullah, Sura Mahmood *et al.* [50]	Deep learning on handwritten records	Handwritten data samples	KNN for classification, genetic algorithms for feature optimization	Exceeded 95% accuracy; demonstrates a reliable technique for PD detection using handwriting analysis
Yang, Mingjing *et al.* [51]	3D ResNet on whole-brain MRI	MRI data	Deep learning for spatial information extraction	Achieved 96.1% accuracy in cross-validation; highlights frontal lobe involvement in PD pathophysiology
Zhang *et al.* [52]	Fractional attribute topology on voice data	Speech samples from different languages	Fractional Fourier transform for feature extraction	Achieved classification accuracies of 99.57%, 95.33%, and 94.13% across diverse datasets, showcasing its effectiveness
Li, Kuan *et al.* [53]	Hybrid DNN on EEG inputs	EEG data from PD patients	CNN and LSTM hybrid architectures for sequential analysis	The parallel model achieved 98.6% accuracy; the series model reached 99.7% accuracy in classifying PD stages
Shcherbak *et al.* [54]	Wearable sensors and ML	Sensor data collected from 113 individuals	ML techniques for feature extraction and classification	Demonstrated f1-micro scores: 0.78 (stage 1), 0.88 (stage 2), useful for non-invasive early PD diagnosis
Ali, Liaqat *et al.* [55]	Ensemble method with feature selection and DNN	Voice data	Integration of deep neural networks with feature selection	Achieved a 6.5% improvement in accuracy compared to traditional methods, demonstrating robust predictive capabilities
Zhao, Aite *et al.* [56]	CNN and bidirectional GRU on handwriting assessments	Handwriting samples (meander, circle, spiral)	Feature extraction *via* CNN; bidirectional GRU for sequence analysis	Achieved identification rates of 92.91%, 85.71%, and 90.55% in respective handwriting tests, highlighting its non-invasive potential
Gupta, Rohan *et al.* [3]	AI and ML in various modalities	Neuroimaging, voice, and gait data	Multimodal analysis with AI techniques	Addressed early detection and patient management through emerging technologies, emphasizing AI's transformative potential in PD care
Hireš, Máté *et al.* [57]	CNNs on voice recordings	Voice recordings of the diagnosed and control groups	Fine-tuning techniques on CNN	Achieved 99% accuracy with specific vowel sounds, offering a practical, non-invasive tool for PD diagnosis and monitoring

**Table 3:** Summary of state-of-the-art methods for epilepsy detection and prediction.

**Study/Refs.**	Methodology	Dataset Used	Computational Techniques	Key Results and Advantages
AbdukodiroV *et al.* [58]	Hyperdimensional Computation	Various	Hybrid models combining individual and general models	Improved model efficiency through information transfer between datasets; applicable in both neuroscience and engineering
Pale *et al.* [59]	BiLSTM with Multi-Head Self-Attention	CHB-MIT	Bidirectional LSTM for seizure detection	Sensitivity: 96.5%, specificity: 97.04%; high accuracy in identifying seizures across multiple patients
Dutta *et al.* [60]	ResCon-LSTM with Dynamic EEG Channel Screening	CHB-MIT	Residual Convolutional Long Short-Term Memory	Achieved 98.523% accuracy; improved detection reliability by dynamically screening EEG channels
Li *et al.* [61]	Dynamic Graph Neural Network	University of Bonn	Graph-based processing with an attention mechanism	Achieved an accuracy of 99.83%, with high precision in leveraging graph properties for EEG classification
Komal *et al.* [62]	Evaluation of Wearable Detection Systems	Various	Non-invasive device analysis	Highlighted performance issues of remote monitoring devices for epilepsy; user acceptance and technology interplay emphasized
Dash *et al.* [63]	Various ML/DL Techniques	Various	SVM, ANN, CNN, and LSTM methods for seizure detection	LSTM-based method achieved 96.5% accuracy; a comprehensive overview of effective methodologies for seizure identification
Ahmad *et al.* [64]	BTBC with SHAP Analysis	Bonn and UCI-EEG	Bagged Tree-based classifier with SHAP for interpretability	Achieved results 2% higher than previous models; improved decision-making processes for diagnosing seizures
Chari *et al.* [65]	Lesion Detection for SEEG	N/A	SEEG-integrated analysis	Enhanced identification of Seizure Onset Zones (SOZ) in children; improves pre-surgical planning in epilepsy treatment
Anita *et al.* [66]	FBSE-EWT and Hybrid Deep Scheme	Traditional Datasets	Fourier-Bessel Series Expansion with CNN and LSTM	Proposed advanced framework for EEG analysis; notable improvements in seizure detection efficiency
Pouryosef *et al.* [67]	Bat and Genetic Algorithms	Various	Multi-parametric feature extraction and classification	Achieved 100% accuracy in balanced classes; effective for clinical EEG analysis with high precision
Basha *et al.* [68]	CGRU-SVM	Bonn University Dataset	Convolutional Gated Recurrent Unit for feature extraction	Achieved an accuracy of 97.54%; highlights the ability to analyze epileptic features through statistical measures
Goel *et al.* [69]	Transfer Learning and Recurrence Graphs	Various	PCA for feature selection and SVM for classification	SVM achieved 98.21% accuracy; emphasizes the importance of feature selection for improving classifier performance
Chen *et al.* [70]	Spiking Conformer	CHB-MIT	Neuromorphic computing with spiking neurons	Specificity: 99.3%, sensitivity: 94.9%; reduces computational load while maintaining high accuracy in seizure detection
Tasci *et al.* [71]	Hypercube Pattern-based Feature Engineering	Large EEG Dataset	Feature extraction through hypercubes	Achieved 87.78% accuracy with voting; highlights the effectiveness of feature engineering for identifying epilepsy
Ilias *et al.* [72]	STFT and Pretrained Models	University of Bonn	Time-frequency analysis with CNN	Efficient feature extraction and multimodal classification using deep learning
Bertoncelli *et al.* [73]	PredictMed-Epilepsy	Longitudinal Multicenter	Machine learning for correlations among clinical factors	Achieved predictive accuracy of 82%; insights into epilepsy management in children with Cerebral Palsy
Lebal *et al.* [74]	Epilepsy-Net	Bonn, Modified Bonn, CHB-MIT	A combination of RNNs and CNNs with an attention mechanism	High accuracies reported: 100%, 99.05%, 98.22%; integration of advanced neural architectures for EEG analysis
Farooq *et al.* [75]	Systematic Review	Various	Overview of feature extraction techniques	Insights on classifier performance and methodologies pertinent to automated seizure detection
Majzoub *et al.* [76]	AlexNet CNN	Various	Convolutional Neural Network for EEG	Achieved binary classification accuracy of 94.44%; significant correlation between dataset selection and accuracy
Al-Hajjar *et al.* [77]	ML and IoMT Integration	Various	Machine Learning and Internet of Medical Things	Emphasized the significance of early seizure detection; enhances remote monitoring and clinical management
Lih *et al.* [78]	EpilepsyNet	Various	Transformer models with Pearson correlation	Demonstrated 85% accuracy, 82% sensitivity, and 87% specificity; effective neurological diagnostics for seizure identification
Saminu *et al.* [79]	Review of AI Techniques	Various	Analysis of AI applications in epilepsy detection	Focus on the integration of deep learning for enhanced application in clinical settings
Shen *et al.* [80]	Tunable-Q Wavelet Transform and CNN	CHB-MIT	Wavelet analysis and CNN for classification	Achieved 97.57% accuracy and 98.90% sensitivity; demonstrates high potential for real-time applications in clinical settings
Qiu *et al.* [81]	DARLNet	Bonn Electroencephalogram Dataset	Hybrid model combining ResNet and LSTM	Addresses previous model deficiencies; effective in distinguishing seizure data within multiple categories
Ahmad *et al.* [82]	Hybrid DL with K-means SMOTE	UCI	1D-CNN and Bidirectional LSTM with SMOTE	Improved precision, sensitivity, and specificity; utilizes unique data-handling techniques to address imbalanced datasets
Miltiadous *et al.* [83]	Systematic Review	Various	Overview of EEG analysis methodologies	Emphasized the growing use of CNN and time-frequency decomposition in seizure detection
Ahmad *et al.* [84]	Review of ML/DL Technology	Various	Feature extraction and machine learning models	Provides recommendations on effective methodologies for seizure detection, addressing strengths and limitations of various approaches
Tawhid *et al.* [85]	ConvLSTM Framework	Various	Convolutional LSTM for EEG analysis	Demonstrates superior performance compared to traditional techniques; effective in automated diagnosis systems
Shoeibi *et al.* [86]	Comprehensive Review	Various	In-depth analysis of DL in seizure prediction	Highlights challenges and future directions for research in epileptic seizure detection and prediction
Sunaryono *et al.* [87]	Gradient Boosting Machines	University of Bonn	Feature extraction with genetic algorithms	Achieved 100% accuracy; demonstrates the effectiveness of GBM fusion methods in epilepsy classification
Tuncer *et al.* [88]	Comparison of DL Methods	Temple University Hospital	Wavelet method and feature selection techniques	Achieved 95.08% accuracy (two-class) and 95.92% accuracy (four-class); efficient in classifying various seizure types
Shen *et al.* [89]	Real-Time EEG Method	UB, CHB-MIT	Eigenvalue algorithms and RUSBoosted tree	Achieved 97% accuracy for real-time detection; emphasizes the effectiveness of continuous monitoring in clinical settings
Forooghifar *et al.* [90]	Self-Aware Wearable System	Lausanne University Hospital	ML techniques for seizure detection	Achieved sensitivity of 88.66% and specificity of 85.65%; highlights improved battery longevity and clinical monitoring capabilities
Jiwani *et al.* [91]	CNN-LSTM Model	Various	Hybrid approach for temporal and spatial analysis	Achieved 100% accuracy; showcases potential for real-time processing in epilepsy diagnosis

**Table 4:** State-of-the-art in huntington's disease research and therapeutics.

**Study/Refs.**	Key Focus	Methodology	Dataset Used	Computational Techniques	Findings	Significance
Paz-Rodríguez F. *et al.* [96]	Cognitive abilities in HD gene carriers	Cognitive testing	HDC cohort (146 participants)	Statistical analysis	The HDC group showed inferior performance in information processing and attention compared to the non-HDC group	Highlights prefrontal cognitive impairment before motor symptom onset in HD patients
Bartl, Stefan *et al.* [97]	Therapeutic potential of monoclonal antibody C6-17	Chronic administration to YAC128 HD mice	YAC128 rodent models	Antibody administration	Reduced HTT protein and improved motor behavior	Demonstrates significant potential of antibody-based treatment for HD
Nicholas Vidas-Guscic *et al.* [98]	White matter abnormalities in HD mouse models	Diffusion MRI (DTI and FBA)	zQ175DN heterozygous mice	DTI and fixel-based analysis	Significant reduction in fiber cross-section/density in the corpus callosum; FBA showed greater sensitivity	FBA metrics as potential noninvasive biomarkers for HD progression
Delussi, Marianna *et al.* [99]	Electrophysiological brain response in HD	MMN recordings, genetic testing, and cognitive evaluations	HD patient group	Event-Related Potentials (ERP) analysis	MMN showed lower global field power in mHD patients	Indicates early functional changes and compensatory mechanisms in the HD phase
Nunes, Adonay S. *et al.* [100]	Speech characteristics in HD	Audio recordings, random forest ML models	Speech data from HD patients and controls	Random forest algorithm	Significant differences in pausing and intonation; AUC of 0.92	Voice data can assess remote illness and monitor HD progression
Jellinger, Kurt A. *et al.* [101]	Cognitive and behavioral changes in early HD	Multimodal imaging, transgenic animal models	Transgenic and clinical samples	Imaging data analysis	Identified degeneration of white/grey matter and synaptic dysfunction	Highlights the need for biomarkers for early diagnosis and treatment
Aldous, Sarah G. *et al.* [102]	Impact of CAG repeat length on HD onset	Genetic analysis, MSH3 gene removal in (CAG)185 mutant mice	CAG repeat mutant mouse models	Genetic manipulation	Reduction in somatic expansion without affecting symptoms	Importance of addressing somatic instability during early disease stages
Sampaio, Cristina *et al.* [103]	Clinical research on HD and HD-ISS	Development of HD Integrated Staging System (HD-ISS)	HD patient cohort	Systematic staging approach	HD-ISS methodically classifies patients prior to diagnosis	Potential for earlier interventions to delay or prevent HD clinical illness
Hassan, Mohamed *et al.* [104]	Microwave sensors for neurodegenerative diseases	Development of an RF antenna	Electromagnetic data from brain imaging	RF signal analysis	Detects minute changes in brain dielectric properties	Non-invasive diagnosis potential for neurodegenerative disorders
Franklin, Gustavo L. *et al.* [105]	Discovery of the HD gene	Genetic mapping and analysis	Blood samples from HD families	Collaborative research methods	Successful identification of the HTT gene	Enabled novel molecular tools and future therapies for HD
Ganesh, Sowmiyalakshmi *et al.* [106]	AI in HD diagnosis	Review of ML and DL techniques	Literature assessment	Machine learning and data analysis	Highlighted trends and challenges in HD diagnosis using AI	Resource for improved HD detection and management
Zhang, Sirui *et al.* [107]	Blood-based biomarkers for HD	Biomarker development	Blood samples	Biomarker analysis techniques	Potential early detection *via* blood biomarkers	Advantages of minimal invasiveness for clinical use
Bains, Rasneer S. *et al.* [108]	Phenotyping HD mouse models	Automated home-cage surveillance	HD mouse model data	Video tracking and activity analysis	Early signs of motor impairment in the HD model	Enhances repeatability in preclinical studies
Stoebner, Zachary A. *et al.* [109]	Cortical abnormalities in HD	MRI studies	HD patient MRI scans	Imaging techniques	Analyzed cortical thickness and gyrification index	May provide clinically meaningful indications of disease progression
Parekh, Neel *et al.* [110]	AI in HD management	Predictive analytics, medical image analysis	Clinical patient data	AI algorithms for predictive modeling	Improved early detection and personalized treatment strategies	Enhances the quality of life for HD patients through AI implementation
Kalra, Jasmeer Singh *et al.* [111]	Predicting HD occurrence	Computer algorithms, mathematical modeling	TRACK-HD dataset and UHDRS features	Machine learning models	Successful prediction of HD progression	Provides precise disease monitoring and prognosis
Alfonso Perez *et al.* [112]	Biomarkers using DNA CpG methylation	Non-linear methods, artificial neural networks	Limited dataset of DNA samples	Machine learning for biomarker identification	Effectively distinguished HD patients from controls	Provides potential for replication with larger datasets
Rosca *et al.* [113]	Cognitive impairment in HD using MoCA	MoCA testing	HD patient population	Sensitivity and specificity analysis	Strong correlation for HD cognitive impairment assessment	Additional research needed for establishing diagnostic thresholds
Podlacha, Magdalena *et al.* [114]	Blood biomarkers in HD mouse models	ELISA assays, mobility, and behavioral assessments	R6/1 rodent model data	Biochemical analysis of blood samples	Identified elevated inflammatory markers linked to cognitive/behavioral symptoms	Highlights biochemical indicators relevant to HD pathology
Paulsen, Jane S. *et al.* [115]	Early HD progression (Predict-HD study)	Non-linear modeling	Predict-HD study cohort	Statistical modeling	Significant changes in biomarkers preceding clinical diagnosis	Advances the understanding of HD etiology and potential biomarkers for prevention
Rosas *et al.* [117]	Neuroimaging methods in HD	Literature review	Various neuroimaging studies	Imaging analyses	Identifies potential neuroimaging as surrogate markers in clinical trials	Addresses challenges in subject availability and assessment sensitivity
Zimbelman, Janice L. *et al.* [118]	Functional brain alterations in pre-HD	Functional MRI studies	Pre-HD and healthy control participant data	fMRI for brain function analysis	Identified functional brain dysfunction up to twelve years before HD onset	Suggests potential for early neuroimaging diagnostics in HD

**Table 5:** Summary of recent advances in ALS research: Diagnostic techniques, biomarkers, and ML approaches.

**Study/Refs.**	Key Focus	Methodology	Dataset Used	Computational Techniques	Findings	Significance
Turner, Martin R. *et al.* [119]	Biomarkers for monitoring ALS progression	Literature review	Various clinical trial datasets	Statistical analysis	Emphasizes the need for sensitive biomarkers to improve diagnostic accuracy and treatment efficacy	Highlights the potential for drug targets through biomarker analysis
Grossman, Alison B. *et al.* [120]	Behavioral changes in ALS patients	Frontal Systems Behavior Scale (FrSBe)	Cohort of 45 ALS patients	Behavioral assessment scoring	Apathy linked to ALS and frontotemporal dementia; FrSBe accurately identifies behavioral issues	Suggests potential for early identification of cognitive changes in ALS
Norel, Raquel *et al.* [121]	Speech analysis for assessing ALS progression	Speech recordings from ALS patients	Prize4Life Israel dataset	Machine Learning models for acoustic analysis	High classification accuracy; monitored progression of ALS through speech	Suggests a non-intrusive assessment method for ALS progression
Wang, Jun *et al.* [122]	ML for ALS diagnosis from brief speech samples	Over 2,500 speech samples from ALS and control patients	ALS speech sample data	Machine learning analysis for speech features	Improved detection performance with articulatory motion info	Demonstrates potential for automation in ALS diagnostics
Zhang, Qi-Jie *et al.* [123]	pTDP-43 aggregation as a biomarker in muscle biopsies	Muscle biopsy samples from ALS patients and controls	54 non-ALS controls and 18 ALS patients	Semi-quantitative scoring of protein aggregation	High sensitivity (94.4%) and specificity (83.3%) for pTDP-43	Suggests potential as an early diagnostic biomarker for ALS
Turco, Antonio *et al.* [124]	Electrochemical biosensor for TDP-43 detection	Lab-on-a-chip system	Serum samples from healthy individuals and ALS patients	Electrochemical impedance spectroscopy	High sensitivity for TDP-43 detection; effective for point-of-care diagnostics	Improves diagnostic capabilities for ALS-related biomarkers
Barel, Dalit *et al.* [125]	Gene panel testing and C9orf72 repeat expansion in ALS patients	Genetic testing cohort	122 ALS patients from Israel	Genetic analysis of C9orf72 and gene panels	Recommendations for genetic testing stratification based on ancestry	Important for personalized treatment approaches in ALS
Simmatis, Leif ER *et al.* [126]	Acoustic speech analysis to categorize ALS severity	Speech samples from ALS patients	119 ALS patients and 22 controls	Sparse Bayesian logistic regression	Successfully categorized ALS severity based on acoustic features	Highlights potential for automated ALS classification
Walter, Uwe *et al.* [127]	Ultrasonography of peripheral nerves in ALS	High-resolution ultrasonography	Cohort of 80 individuals (40 ALS patients, 40 controls)	Imaging analysis of nerve cross sections	ALS patients showed reduced CSA; correlated with respiratory function	Identifies potential functional biomarkers for ALS progression
Darabi, Shahram *et al.* [128]	Review of fluid-based biomarkers	Systematic review of literature	Publications from multiple databases	Literature synthesis	Identified key biomarkers (NFL, TDP-43); emphasizes the need for panel approaches	Provides insights into biomarker utility for diagnosis
Donohue, Cara *et al.* [129]	Assessing ALSFRS-R and Speech Intelligibility Test (SIT) for diagnosing dysarthria	Clinical assessment using ALSFRS-R/SIT	ALS patient cohort	Statistical analysis for sensitivity and specificity	Moderate capacity for dysarthria identification	Confirms the utility of functional scales in ALS diagnosis
Wallace, William *et al.* [130]	Electrochemical detection of phosphorylated TDP-43	Electrochemical biosensor development	Serum samples for testing	Impedance spectroscopy	Effective detection of phosphorylated TDP-43; the method demonstrates selective capability	Innovative approach for ALS biomarker detection
Wu, Yang-Sheng *et al.* [131]	Analysis of ALS progression and related illnesses	Population-based database analysis	ALS patient clinical data	Tree-based modeling	Identified distinct disease progression patterns	Provides insights into ALS mechanisms and risk factors
Christidi, Foteini *et al.* [132]	Hippocampus MRS in non-demented ALS patients	Neuroimaging studies	ALS patient scans	Magnetic Resonance Spectroscopy	Elevated metabolites linked to ALS; potential biomarker for extra-motor disease	Guides future clinical investigations
Udine *et al.* [133]	Identification of ALS-associated genes	Systematic review	Publications from genetic studies	Various sequencing methods and analysis techniques	Highlighted the importance of long-read sequencing for uncovering gene mutations	Enhances understanding of ALS genetics and potential targets for therapy
Vidovic, Maximilian *et al.* [134]	Review of diagnostic procedures and advances in ALS	Literature review	Multiple datasets from ALS studies	Synthesis of recent findings	Advances in biomarkers, imaging, and genetic testing aid early detection	Advocates for improved diagnosis and patient monitoring strategies
Truffert, André *et al.* [135]	Comparison of motor evoked potentials (T-MEPs) in ALS diagnosis	Clinical study	97 ALS patients and 60 matched controls	Electrophysiological assessments	T-MEPs demonstrated high sensitivity (82%) and specificity (93%) for upper motor neuron dysfunction	Validates T-MEPs as reliable diagnostic tools in ALS
Gomes, Nícolas Barbosa *et al.* [136]	Facial expression analysis for early ALS detection	Facial Point Graphs analysis	Toronto Neuroface dataset	Computerized analysis of facial muscle movements	Successfully distinguished ALS patients from controls based on facial movements	Supports the use of automated facial analysis in early ALS diagnostics
Shabber *et al.* [137]	ML for ALS diagnosis using speech recordings	Speech recordings from ALS and control patients	ALS speech dataset	Support Vector Machine (SVM) classification	Achieved a classification accuracy of 98.5% from speech features	Highlights the potential of ML in early ALS identification
Choayb *et al.* [138]	MRI findings in ALS diagnosis	MRI imaging analysis	ALS patient MRI scans	Imaging techniques for structural assessments	Identified characteristic MRI findings supporting ALS diagnosis	Complements clinical electromyography in diagnostic processes
Nishikawa *et al.* [139]	High-density surface electromyography (HD-SEMG) in ALS	Electromyography recordings	16 ALS patients and 16 controls	HD-SEMG analysis	ALS patients showed increased excitability and motor unit abnormalities	Provides insights into neurodegeneration and compensatory motor activity in ALS
Tena *et al.* [140]	Automated speech analysis for diagnosing bulbar involvement in ALS	Acoustic analysis of speech data	Recordings from ALS patients	Random forest classification	Achieved high accuracy (98.01% for females, 96.10% for males); time-frequency features improved prediction	Highlights automated speech analysis as an effective diagnostic tool
Christina N. Fournier *et al.* [141]	Clinical trial design and biomarker validation in ALS	Review of trial methodologies	N/A	Study design analysis	Emphasized patient involvement and biostatistical methods for improved trials	Advocates for comprehensive trial methodologies to enhance ALS treatment outcomes
Mastrogiovanni, Mauricio *et al.* [142]	Plasma oxylipin profiles in ALS and inflammation regulation	Analysis of plasma samples	74 ALS patients and controls	Biochemical assays to evaluate oxylipin levels	Demonstrates altered oxylipin profiles in ALS, indicating inflammation issues	Identifies potential inflammatory biomarkers in ALS pathogenesis
Fukushima, Koji *et al.* [143]	Muscle Ultrasonography (MUS) for fasciculations in ALS diagnosis	Muscle imaging assessments	100 ALS patients and controls	Hierarchical clustering and machine learning techniques	Developed diagnostic models with high sensitivity and specificity for fasciculations	Promising approach for early-stage ALS detection through muscle imaging techniques

## LIST OF ABBREVIATIONS

AD: Alzheimer’s Disease
AI: Artificial Intelligence
ALS: Amyotrophic Lateral Sclerosis
ANN: Artificial Neural Network
AUC: Area Under the Curve
BCI: Brain-Computer Interface
BiLSTM: Bidirectional Long Short-Term Memory
CAG: Cytosine-Adenine-Guanine (nucleotide repeat)
CFS: Correlation-based Feature Selection
CNN: Convolutional Neural Network
COA: Crayfish Optimization Algorithm
CSF: Cerebrospinal Fluid
CSA: Cross-Sectional Area
CV: Cross-Validation
DL: Deep Learning
DTI: Diffusion Tensor Imaging
DT: Decision Tree
DWT: Discrete Wavelet Transform
ECG: Electrocardiogram
EEG: Electroencephalogram
EIS: Electrochemical Impedance Spectroscopy
EMD: Empirical Mode Decomposition
ERP: Event-Related Potential
FBSE-EWT: Fourier-Bessel Series Expansion-Based Empirical Wavelet Transform
FBA: Fixel-Based Analysis
fMRI: Functional Magnetic Resonance Imaging
FrAT: Fractional Attribute Topology
FrSBe: Frontal Systems Behavior Scale
FVC: Forced Vital Capacity
GBM: Gradient Boosting Machine
GFP: Global Field Power
GRU: Gated Recurrent Unit
HD: Huntington’s Disease
HD-SEMG: High-Density Surface Electromyography
HDS: Hybrid Deep Scheme
HET: Heterozygous
IoMT: Internet of Medical Things
KNN: K-Nearest Neighbors
LOD: Limit of Detection
LSTM: Long Short-Term Memory
MAS: Multi-Agent System
MCI: Mild Cognitive Impairment
MDWT: Multilayer Discrete Wavelet Transform
MEG: Magnetoencephalography
ML: Machine Learning
MMN: Mismatch Negativity
MRI: Magnetic Resonance Imaging
MRS: Magnetic Resonance Spectroscopy
MSA-DCNN: Multi-Scale Atrous-based Deep Convolutional Neural Network
MSH3: MutS Homolog 3
MU: Motor Unit
NCA: Neighborhood Component Analysis
NFL: Neurofilament Light Chain
NGS: Next-Generation Sequencing
PD: Parkinson’s Disease
PET: Positron Emission Tomography
pHD: Premanifest Huntington’s Disease
PN: Phrenic Nerve
PTN: Pretrained Neural Network
pTDP-43: Phosphorylated TDP-43
RAVLT: Rey Auditory Verbal Learning Test
RCMDE: Refined Composite Multiscale Dispersion Entropy
RF: Random Forest
RFE: Recursive Feature Elimination
RLDA: Regularized Linear Discriminant Analysis
RNN: Recurrent Neural Network
RMSE: Root Mean Square Error
SAA: Seed Amplification Assay
SEEG: Stereo Electroencephalography
SHAP: SHapley Additive Explanations
SIT: Speech Intelligibility Test
SMOTE: Synthetic Minority Oversampling Technique
SNPs: Single Nucleotide Polymorphisms
SOZ: Seizure Onset Zone
SSA: Salp Swarm Algorithm
STFT: Short-Time Fourier Transform
SVM: Support Vector Machine
TBPTT: Truncated Backpropagation Through Time
TCN: Temporal Convolutional Network
TL: Transfer Learning
UHDRS: Unified Huntington’s Disease Rating Scale
VMD: Variational Mode Decomposition
VN: Vagus Nerve
VGG: Visual Geometry Group
XGBoost: Extreme Gradient Boosting
